# Giant linear plasmids in *Mycobacterium avium* harbour a tRNA array unit

**DOI:** 10.1093/dnares/dsaf039

**Published:** 2026-01-03

**Authors:** Hirokazu Yano, Kentaro Arikawa, Haruo Ikeda, Shouta Nonoyama, Fumito Maruyama, Seigo Kitada, Hiroshi Kida, Manabu Ato, Tomotada Iwamoto, Yukiko Nishiuchi

**Affiliations:** Graduate School of Life Sciences, Tohoku University, Sendai 980-8577, Japan; Antimicrobial Resistance Research Center, National Institute of Infectious Diseases, Japan Institute for Health Security, Higashimurayama 189-0002, Japan; Kobe Institute of Health, Kobe 650-0046, Japan; Technology Research Association for Next Generation Natural Products Chemistry, Tokyo 135-8073, Japan; Graduate School of Life Sciences, Tohoku University, Sendai 980-8577, Japan; Center for Planetary Health and Innovation Science, the IDEC Institute, Hiroshima University, Higashihiroshima 739-8530, Japan; National Hospital Organization Osaka Toneyama Medical Center, Toyonaka 560-8552, Japan; National Hospital Organization Osaka Toneyama Medical Center, Toyonaka 560-8552, Japan; Leprosy Research Center, National Institute of Infectious Diseases, Japan Institute for Health Security, Higashimurayama 189-0002, Japan; Kobe Institute of Health, Kobe 650-0046, Japan; Center for Planetary Health and Innovation Science, the IDEC Institute, Hiroshima University, Higashihiroshima 739-8530, Japan; Toneyama Institute for Tuberculosis Research, Osaka City University Graduate School of Medicine, Toyonaka 560-8552, Japan

**Keywords:** nontuberculous mycobacteria, invertron, linear plasmid, T7SS, *Mycobacterium avium* complex

## Abstract

Nontuberculous mycobacteria occasionally harbour clustered tRNA genes, referred to as a tRNA array unit, which is considered a putative antidefense system within their genomes. However, the precise genomic location of these tRNA array units remains unclear. To address this, we sequenced the complete genomes of 5 *Mycobacterium avium* strains carrying a tRNA array unit using a hybrid assembly of long and short reads followed by manual curation. The assemblies indicated that each strain harbours 3 to 5 extrachromosomal elements. In all genomes, the tRNA array unit was found on a linear contig exceeding 300 kb. Pulse-field gel electrophoresis (PFGE) and sodium dodecyl sulphate-PFGE revealed that the strains harbour linear plasmids corresponding to these large contigs with protein-capped termini. These linear plasmids encode a hybrid type VII/type IV secretion system but lack relaxase genes, which are typically present in mycobacterial circular plasmids. Additionally, they contain approximately 415 bp inverted repeats at the termini. Sequences of related plasmids were identified exclusively in the genomes of *M. avium* isolates from Japan available in public databases, suggesting a possible Asian origin. This study provides the first experimental evidence that *M. avium* harbours giant invertron-type linear plasmids carrying a tRNA array unit.

## 1. Introduction

Nontuberculous mycobacteria (NTM) are opportunistic human pathogens capable of causing chronic pulmonary diseases, even in immunocompetent individuals.^1^ NTM pulmonary diseases are notoriously difficult to treat and often require prolonged chemotherapy, placing a significant burden on affected patients.^1^ NTM are widely distributed in the environment, and cumulative exposure to water or soil is considered a risk factor for NTM-associated pulmonary diseases.^2^ The global incidence of these diseases is currently increasing.^2^ Clinically, the most frequently isolated mycobacterial species worldwide belong to the *M. avium* and *M. abscessus* complexes.^2,3^ Among these, *M. avium* is the most common in respiratory specimens across several countries and regions, where comprehensive epidemiological and bacteriological data are available.^2,3^

*M. avium* is classified into 3 established subspecies—subsp*. paratuberculosis*, subsp*. silvaticum*, and subsp*. avium—*as well as one provisional subspecies—subsp. *hominissuis.*^4^  *M. avium* isolates from patients with chronic lung disease belong to subsp. *hominissuis.*^4^ Comparative genomic studies of subsp. *hominissuis* have revealed that intraspecies chromosomal recombination is common in some lineages. Among isolates derived from human infections, specific lineages are associated with the geographic regions from which the isolates were obtained.^5,6^ These patterns show partial similarity to those observed in *M. abscessus*.^7^ In the case of *M. abscessus*, several clones (termed dominant circulating clones)—which are thought to be more adapted to pulmonary infections—have been reported to spread globally in clinical settings.^8,9^ However, such clonal expansion has not been documented in *M. avium*.

To date, complete genome sequences of several *M. avium* strains have been reported.^5,10,11^ Notably, Dragset *et al.*^12^ identified strain MAH 11 as susceptible to TM4 phage infection, sequenced its complete chromosome, and subsequently developed a phage-mediated high-throughput transposon mutagenesis and sequencing technique, known as Tn-Seq, using this strain to identify a set of genes involved in infection in mice.^12^ Similarly, Matern *et al.*^11^ reported that *M. avium* strain MAC 109 exhibits high susceptibility to TM4 phage infection. Furthermore, they completed its genome sequencing and developed Tn-Seq for this strain.^13,14^ While the development of Tn-Seq using strains MAH 11 and MAC 109 has significantly advanced the bacteriological study of *M. avium*, these strains belong to lineage SC3, and SC4, respectively,^6^ which are not representative of the major lineages of *M. avium.* The gene repertoire of *M. avium* is extremely diverse^15^; hence, phenotypic traits such as antimicrobial resistance, biofilm formation, and infectivity are expected to be diverse across subspecies and lineages. Thus, expanding the availability of complete genome sequences from strains representing diverse lineages is essential to advance research on *M. avium*.

Plasmids are defined as autonomously replicating extrachromosomal elements. Although generally nonessential, they can carry genes that confer adaptive traits to bacterial cells, such as antimicrobial resistance.^16,17^ In Gram-positive bacteria, plasmids may harbour gene clusters responsible for polyketide biosynthesis.^18–20^ Although the widely studied *M. avium* strain 104 lacks plasmids, plasmid occurrence seems to be common in *M. avium,* as suggested by pulse-field gel electrophoresis (PFGE) on clinical isolates^21,22^ and a recent data mining of public databases.^23^ However, PFGE and long-read sequencing have not been performed for most reported *M. avium* strains, resulting in limited literature on *M. avium* plasmids. Uchiya *et al*.^22^ suggested that the plasmid pMAH135 may contribute to virulence due to its carriage of genes involved in polyketide biosynthesis, potentially for mycobactin production. Hence, more annotated genomes, including plasmid sequences, are needed to improve our understanding of *M. avium*.

**Fig. 1.**
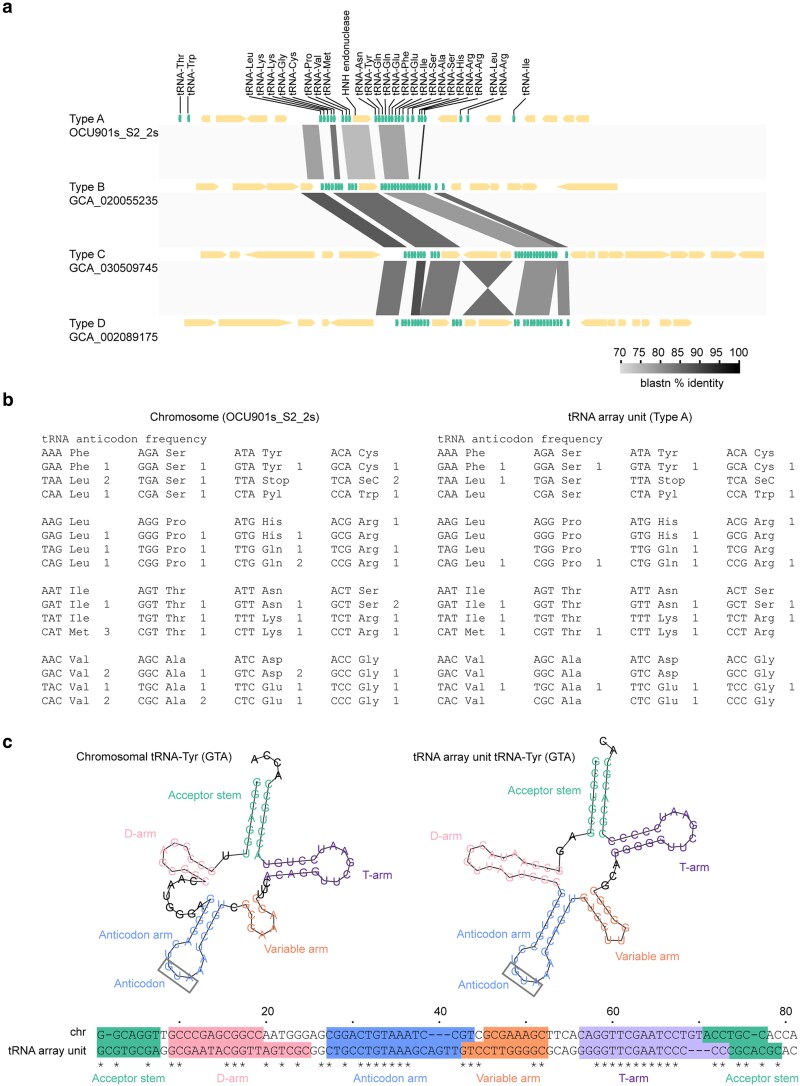
(a) Four types of tRNA array units identified in *M. avium* subsp. *hominissuis*. In the gene maps, protein-coding sequences and tRNA genes are represented in light yellow and green, respectively. Type A is associated with pS2b-type linear plasmids analysed in this study. Sequences were obtained from the following RefSeq/Genomes accession numbers: Type A, CP076860.2 (pS2b); Type B, GCA_020055235, contig 1 (*M. avium* strain GM17); Type C, GCA_030509745, contig 22 (*M. avium* strain FLAC1199); Type D, GCA_002089175, contig 17 (*M. avium* strain DH-6). (b) Anticodons represented by tRNAs encoded on the chromosome contig and within the Type A tRNA array unit in the genome of OCU901S_S2_2s. (c) Nucleotide sequences of tRNA-Tyr-GTA gene and its variant encoded within the Type A tRNA array unit. The predicted tRNA secondary structures are shown above the sequences.

As of November 2024, 189 genomic assemblies of *M. avium* subsp. *hominissuis* strains have been released in GenBank. All *M. avium* genome assemblies commonly harbour a single rRNA operon, whereas 15 genomes exhibits an unusually high number of tRNA genes (over 70 compared to the median of 55, Table S1 excluding strains BA018, BA059, BA705, and PPE_S58). In most assemblies, the additional set of tRNA genes that cover codons for 19 to 20 amino acids, is clustered within a single contig. This tRNA gene cluster has been previously designated as a tRNA array unit (Fig. 1).^24^ Although the biological role of the tRNA array unit is unknown, studies of defence systems in nonmycobacterial genomes suggest that these clustered tRNA genes in the array unit may function as an antidefense mechanism.^25,26^ The tRNA array unit has been identified not only in *M. avium* draft genomes but also in other mycobacterial genomes, mycobacteriophages, and plasmid genomes.^24^ However, the precise genomic location of the tRNA array unit within *M. avium*—whether on the chromosome, plasmid, or mycobacteriophage—remains unknown due to the limited availability of complete genome sequences of *M. avium*.

In this study, to advance our understanding of the genome organization in *M. avium*, we generated complete genome sequences of 5 *M. avium* subsp. *hominissuis* strains from our laboratory collection, all of which harbour a tRNA array unit. Two strains were isolated from patients with MAC lung disease, while 3 originated from bathrooms in the households of healthy volunteers. The combined results of genome assembly and electrophoresis analyses showed that the tRNA array unit was located on a linear plasmid in all 5 strains, indicating its potential biological role in the horizontal gene transfer of these linear plasmids. Furthermore, we identified a 153-kb genomic island on one linear plasmid, which is predicted to be involved in lipid metabolism and shares a substantial gene composition with a segment of a circular plasmid from *M. paragordonee*. This finding suggests the translocation of large DNA segments among NTM plasmids.

## 2. Materials and methods

### 2.1. Strains and medium

The *M. avium* strains used in this study were OCU901s_S2_2s, PPE_S58, BA018, BA059, and BA705. The OCU901s_S2_2s and PPE_S58 strains were isolated from patients attending the National Hospital Organization, Osaka Toneyama Medical Center. Strain OCU901s_S2_2s was isolated in 2007 from a patient with no history of chemotherapy and diagnosed with nodular bronchiectasis. Strain PPE_S58 was isolated in 2011 from a patient with a history of chemotherapy who also presented with nodular bronchiectasis. Strains BA018, BA059, and BA705 were obtained from bathtub inlet biofilms in the bathrooms of healthy volunteers’ households, as reported in our previous study.^27^ Specifically, BA705 was isolated in 2019 from a household in Habikino City, while BA018 and BA059 were collected in 2017 from households in Kobe City. *E. coli* strain DH5α (NIPPON GENE CO., LTD.) was used for DNA cloning. A long-read only assembly of the OCU901s_S2_2s genome was published in our previous study.^5^

*M. avium* strains were cultured on BD Difco^TM^ MiddleBrook 7H10 agar supplemented with 10% BD BBL^TM^ Middlebrook OADC growth supplement for 2 to 3 wk at 37 °C. *M. avium* liquid culture was prepared in BD Difco MiddleBrook 7H9 broth supplemented with 10% BD BBL Middlebrook ADC growth supplement, 0.2% glycerol, and 0.05% Tween 80. *E. coli* strain was cultured in Luria–Bertini Lennox broth and its agar medium supplemented with 1.5% agar and ampicillin at 100 μg/mL.

### 2.2. Pulse-field gel electrophoresis and sodium dodecyl sulphate-PFGE

PFGE and sodium dodecyl sulphate (SDS)-PFGE were performed using *M. avium* liquid cultures grown to a McFarland standard of 0.7 to 1.0 in Middlebrook 7H9 broth supplemented with 10% ADC enrichment and 0.2% Tween-80. A 0.5 mL aliquot of the culture was centrifuged. The cells were resuspended in 400 μL of 1.6% chromosomal-grade agarose and then solidified in Bio-Rad 50-well disposable plug moulds (Bio-Rad, Hercules, CA, USA). Eight to 10 plugs were digested in 5 mL of lysis buffer [1 M NaCl, 10 mM Tris-HCl (pH 8.0), 100 mM EDTA, 0.5% Sarkosyl, 0.5% Brij 58, 0.2% sodium deoxycolate] containing 10 mg/mL lysozyme and incubated in a 13 mL tube at 37 °C for 24 h. Subsequently, 4 to 5 plugs were transferred to 2 mL of freshly prepared proteinase K solution [2 mg/mL proteinase K, 1% N-laurolyl sarcosine, 0.5 M EDTA] and incubated at 55 °C for 24 h. Electrophoresis was performed using a Bio-Craft BS-80 Pulse-Field Gel Electrophoresis System (Bio-Craft, Tokyo, Japan). The agarose gel was prepared with 1.1% SeaKem Gold Agarose (Lonza, Tokyo, Japan) in 0.5× TBE buffer containing 50 μM thiourea. Electrophoresis conditions were 180 V, 15 °C, at a constant pulse switching interval of 15 s for 24 h (for strains OCU901s_S2_2s, BA705, and BA059) or 26 h (for strains PPE_S58 and BA018). For SDS-PFGE, 0.1% SDS was added to the 0.5× TBE buffer. A heat-treated lambda ladder (CHEF DNA size standards, Bio-Rad, Hercules, CA, USA) was used as a size marker.

### 2.3. Genomic DNA extraction and sequencing

Genomic DNA for next-generation sequencing (NGS) was prepared using one of 2 bead-beating methods, one with proteinase K treatment and the other without it. In the beads-beating method with proteinase K treatment, *M. avium* cells were suspended in 2-mL screw-cap tubes containing 250 μL of Az One BZ-02 glass beads (As One Corporation, Osaka, Japan) and 750 μL of TE buffer (10 mM Tris-HCL, 1 mM EDTA, pH 8.0). The tubes were vortexed in a bead beater Micro Smash™ MS-100R (TOMY SEIKO, Co. Ltd., Tokyo Japan) at 2,000 rpm for 1 min. Following bead beating, 500 μL of phenol/chloroform/isoamyl alcohol (PCI, 25:24:1) was added, and the tubes were inverted thoroughly before centrifugation at 15,000 rpm for 5 min. The aqueous phase was transferred to 1.5-mL tubes, treated with 2.5 μL of RNase A at a final concentration of 20 μg/mL, and the mixtures were incubated at 37 °C for 30 min. Proteinase K was then added to the mixture at a concentration of 200 μg/mL and incubated overnight at 50 °C. The following day, an additional 500 μL of PCI was added, and the samples were centrifuged again at 15,000 rpm for 5 min. The aqueous phase was transferred to new tubes, and the DNA was precipitated by ethanol precipitation and rinsed with 70% ethanol before being resuspended in 100 to 200 μL elution buffer (10 mM Tris-HCl, pH 8.5). Genomic DNA was prepared without proteinase K treatment at the Kobe Institute of Health, following a similar protocol, except the proteinase K treatment step was omitted, PCI was added before bead beating, and bead beating was conducted using a Vortex-GENIE 2 device (Scientific Industries, Inc., NY, USA).

Long-read sequencing data for strain OCU901s_S2_2s were generated using proteinase K-treated genomic DNA on the PacBio RS II platform.^5^ Conversely, long reads for strains PPE_S58, BA018, BA705, and BA059 were obtained from proteinase K-untreated DNA and sequenced on the PacBio Sequel II platform at the University of Minnesota Genomics Center (MN, USA). Illumina NGS libraries were prepared from proteinase K-treated genomic DNA using the TruSeq DNA PCR-Free Kit (Illumina Inc., San Diego, CA, USA). Paired-end reads for OCU901s_S2_2s were obtained from the HiSeq 2500 platform at Macrogen Japan (Tokyo, Japan), while those for PPE_S58, BA018, BA059, and BA705 were obtained using the NovaSeq 6000 platform at NovogeneAIT Genomics (Singapore).

### 2.4. Genome assembly

Genome assembly was performed using Unicycler (v.0.4.8) with default parameters for hybrid assembly.^28^ Before assembly, short reads were quality-trimmed using Rfastp (v0.21.0)^29^ with the options ‘cutLowQualTail = TRUE’ and ‘cutTailMeanQual = 30’. The Unicycler outputs for all 5 strains contained a single linear contig, which was presumed to represent incomplete sequences of linear replicons. To complete the assemblies, Illumina short reads overlapping these linear contigs were further identified and incorporated. All assemblies were polished using Pilon (v.1.23) with short reads.^30^

#### 2.4.1. Linear contig of OCU901s_S2_2s

Trimmed short reads were first mapped to the Unicycler hybrid assembly using Bowtie2 (v2.5.1) with options ‘-q -N 1 -S’,^31^ and unmapped reads were extracted using samtools (v1.13) with ‘bam2fq -f 4’.^32^ Reads overlapping the linear contig ends were identified using BLASTN (v2.16.0+),^33^ and paired-end reads—either of which overlapped with linear contig termini—were retrieved using SeqKit with the “grep -f” option.^34^ Reads starting with a poly-C tract (up to 9 C nucleotides on one end and 8 on the other end) overlapped with either end of the linear contig. These reads were attached to the linear contig as overhangs to finalize the linear contig assembly, yielding a 348,162 bp complete sequence of the linear replicon pS2b. This assembly was validated through restriction fragment length analysis of proteinase K-treated total DNA, followed by Southern hybridization using the terminal regions as probes (Figure S1).

#### 2.4.2. Linear contig of BA018

Following a similar strategy for the OCU901s_S2_2s assembly, trimmed short reads were mapped to the Unicycler hybrid assembly, and unmapped reads overlapping with either end of the linear contig were identified and retrieved. Paired-end reads containing a fragment of either end of the linear contig were merged using fastp^29^ with the ‘-m’ option. Two merged reads with poly-C tracts at their 5′ ends were appended as overhangs to either end of the linear contig, yielding a 366,138-bp complete sequence of linear replicons designated as pBA018a.

#### 2.4.3. Linear contig of PPE_S58

In addition to the Unicycler hybrid assembly, a short-read-only assembly was generated using untrimmed short reads with the Shovill pipeline (default setting).^35^ Among the contigs obtained by Shovill assembly, 6,500 and 14,226 bp sequences starting with poly-C tracts at their 5′ ends overlapped the termini of the Unicycler linear contig. These contigs were attached as overhangs, yielding a 456,679-bp complete sequence of the linear replicon pPPE_S58a.

#### 2.4.4. Linear contig of BA705

Following the approach used for PPE_S58, additional short-read-only assemblies were generated. The linear contig from the hybrid assembly was extended in both directions by merging it with a 21,157-bp contig from the short-read-only assembly, which contained a poly-C sequence at one end and a 30,254-bp contig without a poly-C sequence. The estimated 20 bp missing segment at one linear replicon end was insufficiently covered by short reads but was presumed to mirror the other end of the linear replicon. The missing sequence was identified in the raw reads using the sequence motif search function of the SeqKit (‘grep -s -p’). Paired reads containing the motif were merged and appended as an overhang to one linear contig. The final assembly was polished, and a 308,290-bp complete sequence of the linear replicon pBA705a was obtained.

#### 2.4.5. Linear contig of BA059

Similar to the PPE_S58 linear contig finishing, a linear contig from the hybrid assembly was first extended in both directions by merging with 24,751-bp and 3,312-bp contigs without poly-C nucleotide ends, which were obtained from short-read-only assemblies. Terminal sequence motifs of the extended contig were searched in the raw short reads using the SeqKit (‘grep -s -p’). Reads containing both the motif and poly-C nucleotide end were identified; their low-quality 3′ ends (quality value < 12) were trimmed before appending the trimmed single-end reads as overhangs to the extended linear contig. The final assembly was polished, yielding a near-complete linear replicon sequence of 344,653 bp, designated as pBA059a.

Except for the circular contig cS2d from strain OCU901s_S2_2s, all circular contigs generated by the Unicycler pipeline harboured at least one putative plasmid-associated gene (eg a relaxase gene) and were therefore regarded as extrachromosomal elements. In the short-read-only Shovill assembly, the sequence corresponding to contig cS2d was embedded within a gene encoding heavy metal—translocating P-type ATPase located on a 321-kb contig that matched the chromosomal contig obtained from the hybrid assembly. We inferred that the separation of contig cS2d from the chromosomal contig in the hybrid assembly likely resulted from recombination between duplicated genes on the original chromosome in the cell population used for long-read sequencing library preparation. Therefore, the sequence of cS2d was submitted to the database but was not treated as a plasmid sequence in this study.

Plasmid copy number was calculated as the ratio of the median mapped short-read coverage of the plasmid to that of the chromosome, determined using the ‘genomecov-bg’ function of bedtools v2.31.1.^36^

The folding of 3′ ends of the linear replicons was predicted at 37 °C and 1 M Na^+^ using the mFold web server.^37^

### 2.5. Southern hybridization

A segment from the left end region of pS2b was PCR-amplified using OneTaq Hot Start 2× Master Mix (New England Biolabs, Inc., Ipswich, MA, USA) with the primer pair pS2_right3_F (5′-GGCCACAAAGTGATTGGAGT-3′) and pS2_right3_R (5′-TCGATGCTGTATTCGGTGAA-3′). The PCR product was cloned into the pGEM-T vector using the pGEM-T Vector System (Promega, Madison, WI, USA), yielding the plasmid designated pHY1362. Similarly, a segment from the right end region of pS2b was PCR-amplified using the primer pair pS2_left_F (5′-GGCTTGGTTATGGCGAACTA-3′) and pS2_left_R (5′-GTCGGAGCGTAAA GCAAGAC-3′), and cloned into the pGEM-T vector, yielding the plasmid designated pHY1363. The inserts from pHY1362 and pHY1363 were PCR-amplified using the same primer sets and purified. The resulting PCR products were labelled with digoxigenin (pHY1362 insert) or biotin (pHY1363 insert) using the Invitrogen BioPrime Array CGH Genomic Labeling System (Thermo Scientific, Waltham, MA, USA), along with Digoxigenin-11-dUTP and Biotin-16-dUTP (Roche, Basel, Switzerland). These labelled products served as the digoxygenin-labelled left-end probe and the biotin-labelled right-end probe of pS2b, respectively.

Proteinase K-treated genomic DNA from OCU901s_S2_2s for Southern hybridization was electrophoresed in a 0.8% agarose gel in 0.5× TAE for 99 min at 135 V. The DNA was then transferred to a BioDyne Plus membrane (Pall Corporation, NY, USA) by capillary blotting following the manufacturer’s recommended protocol. The membrane was then incubated at 65 °C using PerfectHyb Hybridization Solution (Toyobo, Co., Ltd., Osaka, Japan) without the addition of SDS. Hybridization was performed using the following reagents: IgG Fraction Monoclonal Mouse Anti-Digoxin (Jackson ImmunoResearch, Inc., West Grove, PA, USA), IRDye 680LT goat anti-mouse IgG secondary antibody (LI-COR Biosciences, Lincoln, NE, USA), and IRDye 800CW streptavidin (LI-COR Biosciences) in PerfectHyb. The membrane was washed according to the manufacturer’s instructions (Toyobo, Co., Ltd.) and scanned using the LI-COR Odyssey imaging system (LI-COR Biosciences).

### 2.6. Detection and grouping of the ESX locus

RefSeq protein sequences and GFF3 annotation files for complete genomes classified under the order Mycobacteriales (as of 1 November 2024; 1,613 genomes) were retrieved from the NCBI Datasets Genome database (https://www.ncbi.nlm.nih.gov/datasets/genome/) using the following filters: ‘Mycobacteriales’, ‘complete’, and ‘annotated by NCBI RefSeq’. We defined the ESX locus as a genomic region of <30 kb harbouring both *eccB* and *eccD* or a region harbouring both *eccB* and *eccA*. Homologues of EccA, EccB and EccD were identified from the protein datasets of the 1,613 genomes using the PHMMER function of HMMER v3.4^38^ based on E-value cutoff 0.01. pS2b EccA, EccB and EccD sequences were used as initial queries. The second round of PHMMER search was performed for EccD using the most distant hit (protein ID WP_163749646) as a new query. A maximum-likelihood phylogenetic tree of the ESX loci was constructed using IQ-TREE 2 with a nonreversible substitution model,^39^ based on EccB homologue sequence alignments generated using MAFFT (v7.407) with the L-INS-i algorithm.^40^ Gene synteny comparisons were conducted using GenomeMatcher.^41^ Phylogenetic trees and their metadata were visualized using R package ggtree.^42,43^

### 2.7. Phylogenetic tree inference for *M. avium* subsp. *hominissuis*

Publicly available genomes of *M. avium* subsp. *hominissuis* were retrieved from the NCBI Datasets portal (https://www.ncbi.nlm.nih.gov/datasets/) as of 11 November 2024, using the ‘GenBank’ filter. This dataset comprised 189 genomes, excluding one entry that contained only plasmid data. The 5 newly sequenced genomes from this study were added to this dataset, resulting in a total of 193 genomes. To infer the chromosomal phylogeny of these genomes, high-quality single-nucleotide polymorphisms (SNPs) were identified using Snippy,^44^ with the chromosome of strain TH135 (GenBank accession number AP012555.1) used as the reference. Chromosomal sequence alignments encompassing 60,546 polymorphic sites were generated using an in-house R script based on VCF files that were generated using Snippy. Phylogenetic inference was performed using ClonalFrameML, a recombination-aware method.^45^ The initial tree was constructed using chromosomal sequence alignment and the GTR model in IQ-TREE 2.^39^

### 2.8. Gene composition analysis of mycobacterial plasmids

Plasmid sequences associated with the family Mycobacteriaceae, hereafter referred to as NTM plasmids, were retrieved from the NCBI Nucleotide database (https://www.ncbi.nlm.nih.gov/nucleotide/) using the following filters: ‘RefSeq’, ‘Plasmid’, ‘Mycobacterium’, and a sequence length range of 10,000 to 1,000,000 bp. The retrieved sequences were further filtered based on taxonomic classification and merged with the plasmid sequences determined in this study, resulting in a dataset comprising 314 NTM plasmids (as of 18 May 2025). All plasmid sequences were annotated in-house using DFAST-core^46^ with default parameter settings. Gene families and alleles in the dataset were identified using the GFF annotation files produced by DFAST-core and analysed with the PRITATE pangenome analysis pipeline,^47^ using the ‘-s 50,60,70,80’ option. For this study, all protein clusters split at a 50% to 80% identity threshold were treated uniformly as gene families. The PIRATE output file ‘PIRATE.gene_families.ordered.tsv’ was converted to a binary matrix file using the ‘PIRATE_to_Rtab.pl’ script with the ‘−l 0’ option to perform hierarchical clustering of NTM plasmids based on gene composition dissimilarity. The NTM plasmids are listed in Table S3. Pangenome analysis was performed for the dataset containing only the 5 linear plasmids and for another dataset containing the same 5 plasmids along with 9 additional draft genomes harbouring both tRNA array unit and pS2b-type *eccB*, using PIRATE under the same parameter setting as described above.

### 2.9. Identification of insertion sequences

Insertion sequences (ISs) in the genomic assemblies were identified using BLASTN searches in the ISfinder database.^48^ Genomic regions with hits exhibiting a bit score >500 and a sequence length >500 bp were classified as IS or inactivated IS copies.

### 2.10. Annotation

Genome annotations were routinely performed using the standalone version of DFAST (DFAST-core v1.3.6).^46^ Homologues of Cluster of Orthologous Genes (COGs), as defined by NCBI,^49^ along with their associated functional categories, were identified through the DFAST-core. Homology searches were conducted using either BLAST+^33^ or HMMER v3.4.^38^

### 2.11. Detection of tRNA array units in genomes

The tRNA genes were annotated from FASTA files using ARAGORN v1.2.38^50^ with the ‘-fo’ option. The tRNA array units were identified from the ARAGORN output using a Perl script provided by Mordado and Vicente.^24^ Secondary structure of tRNA was predicted using RNAfold web server.^51^

## 3. Results and discussion

### 3.1. tRNA genes in the tRNA array unit

tRNA array units identified in *M. avium* subsp. *hominissuis* from the NCBI Genome Database were classified into 4 types, provisionally termed Type A through Type D (Fig. 1a), based on the composition of their tRNA genes. The different tRNA array units exhibited 77% to 89% nucleotide identities within the BLASTN-aligned regions. Each unit contained 26 to 27 tRNA genes collectively corresponding to codons for 19 to 20 amino acids. Among the 4 variants, Types B, C, and D were rare in *M. avium* (Table S1). The flanking regions of these 4 types of array units showed no sequence similarity, indicating that they are inserted at distinct genomic locations in *M. avium* draft genomes. A single HNH endonuclease–encoding gene embedded within the array unit was conserved across all 4 types. The anticodons of tRNAs derived from the array unit were redundant with those of chromosomally encoded tRNAs (Fig. 1b). Therefore, the tRNA genes within the array unit are likely nonessential for bacterial growth. The nucleotide sequences differed between each chromosomally encoded tRNA and its corresponding tRNA from the array unit among all redundant tRNAs. Figure 1c illustrates an example of tRNA-Tyr. Structural differences were typically observed in the D-arm, anticodon arm, and variable arm—collectively referred to as the ‘*anticodon domain*’—in the predicted tRNA folds.^52^ Whether the tRNA gene variants in the array unit retain functional roles related to translation or antidefense remains to be determined.

Prior to this study, we performed either short-read-only or long-read-only sequencing of *M. avium* subsp. *hominissuis* isolates collected at the Kobe Institute of Health and identified tRNA array units in the draft genomes of 43 out of 189 strains (unpublished data). In this study, we focused on 5 tRNA array unit-positive strains—OCU901s_S2_2s, BA018, PPE_S58, BA059, and BA705—to generate reference-grade complete genome assemblies.

### 3.2. End-to-end sequences of *M. avium* linear replicons

For OCU901s_S2_2s, we generated new Illumina short reads to complement previously obtained PacBio RSII long reads. For the remaining 4 strains, we generated Illumina short reads and PacBio Sequel II HiFi long reads. All NGS libraries were prepared using a PCR-free method. Using the Unicycler hybrid assembly pipeline, we generated 3 to 4 circular contigs and one linear contig per strain (Table 1). In 4 of the 5 strains, a circular contig larger than 190 kb, which was highly similar to the previously reported polyketide biosynthesis plasmid pMAH135,^22^ was identified and designated as pS2a, pPPE_S58b, pBA059b, or pBA705b. In all 5 strains, the linear contigs harboured a tRNA array unit comprising 26 to 27 clustered tRNA genes.

**Table 1.** Genomic organization of 5 *M. avium* subsp. *hominissuis* strains.

Strain	Molecule name^a^	Size (bp)	Relative copy number (per chromosome)	G+C %	No. of rRNA gene	No. of tRNA gene	No. of CDS	GenBank accession number	Notable features and genes
OCU901s_S2_2s	Chromosome	5,182,265	1.0	69.1	3	55	4,850	AP041818	MahEastAsia2
	pS2b	348,162	1.3	64.2	0	27	418	AP041819	linear topology, tRNA array unit, ESX-P5
	pS2a	194,253	0.9	66.5	0	1	162	AP041820	pMAH135-type polyketide synthesis plasmid, ESX-P1, MobF family relaxase
	pS2c	12,893	2.0	66.4	0	0	16	AP041822	MobF family relaxase
	cS2d^b^	16,887	0.9	64.5	0	0	16	AP041821	Circular contig. Originally part of the chromosome
PPE_S58	Chromosome	5,155,927	1.0	69.1	3	55	4,824	AP038890	MahEastAsia2
	pPPE_S58a	456,679	1.5	64.5	0	27	528	AP038891	linear topology, tRNA array unit, *menB*, *lpqH*, ESX-P5, MCE operon
	pPPE_S58b	199,950	1.0	66.5	0	1	167	AP038892	pMAH135-type polyketide synthesis plasmid, ESX-P1, MobF family relaxase
	pPPE_S58c	12,893	0.9	66.4	0	0	15	AP038893	MobF family relaxase
BA018	Chromosome	5,355,123	1.0	69.0	3	53	5,087	AP038875	MahEastAsia1
	pBA018a	366,138	1.0	64.4	0	26	441	AP038876	linear topology, tRNA array unit, ESX-P5
	pBA018b	82,401	1.8	65.9	0	0	86	AP038877	ESX-P3, MobF family relaxase
	pBA018c	21,577	1.2	65.3	0	0	20	AP038878	MobF family relaxase
BA059	Chromosome	5,064,937	1.0	69.1	3	54	4,749	AP038879	MahEastAsia2
	pBA059a	344,653	1.3	64.4	0	27	427	AP038880	linear topology, tRNA array unit, ESX-P5
	pBA059b	195,724	1.0	66.5	0	1	163	AP038881	pMAH135-type polyketide synthesis plasmid, ESX-P1, MobF family relaxase
	pBA059c	101,695	1.6	66.0	0	0	103	AP038882	ESX-P1, MobF family relaxase
	pBA059d	82,370	1.4	65.9	0	0	86	AP038883	ESX-P3, MobF family relaxase
	pBA059e	15,433	1.6	64.8	0	0	15	AP038884	MobF family relaxase
BA705	Chromosome	5,217,390	1.0	69.0	3	55	4,896	AP038885	MahEastAsia2
	pBA705a	308,290	1.5	64.4	0	27	389	AP038886	linear topology, tRNA array unit, ESX-P5
	pBA705b	200,636	1.0	66.5	0	1	169	AP038887	pMAH135-type polyketide synthesis plasmid, ESX-P1, MobF family relaxase
	pBA705c	87,665	1.8	65.5	0	0	102	AP038888	ESX-P3, MobF family relaxase
	pBA705d	12,893	3.0	66.4	0	0	16	AP038889	MobF family relaxase

^a^DNA molecules other than linear plasmids were identified based on Unicycler assembly. Their topologies were estimated to be circular.

^b^cS2d is a circular contig generated by Unicycler hybrid assembly pipeline and is presumed to be formed by homologous recombination between 2 directly oriented copies of a heavy metal–translocating P-type ATPase-encoding gene located in the chromosome (AP041818 coordinate: 3,021,818-3,023,29).

**Fig. 2.**
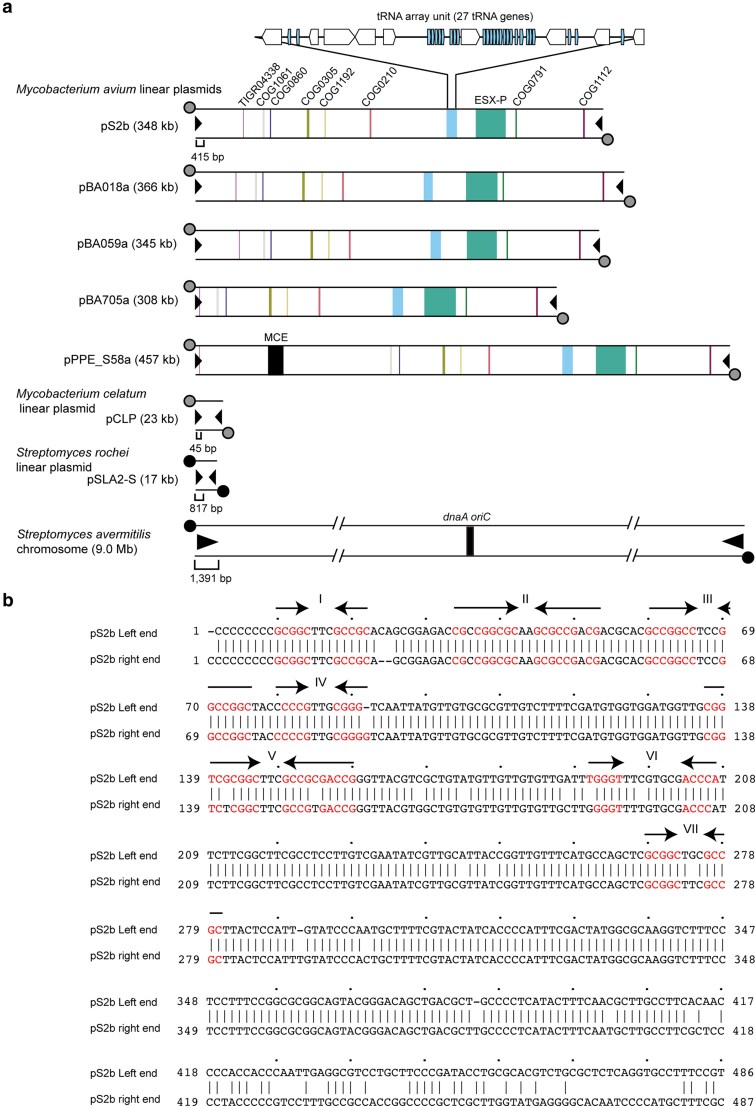
(a) Schematic representation of the structure of linear *M. avium* plasmids. Locations of representative core genes are indicated by vertical lines labelled with COG or TIGERFAM identifiers. The *parA* gene is annotated as COG1192. Genes encoding peptidases include TIGR04338, COG0860, and COG0791. DNA/RNA helicase-encoding genes are represented by COG0305, COG0210, COG1112, and COG1061. Grey circles indicate putative (unknown) terminal proteins, while black circles represent characterized (known) terminal proteins. (b) Near-complete terminal region sequences of plasmid pS2b. Predicted folding of the single-stranded 300 nt 3′ terminal region of DNA was obtained using mfold.^37^ Regions forming stem loops in complementary strands are indicated by arrows and numbered using Roman numerals. Terminal sequences of the other 4 linear plasmids and pCLP are shown in Figure S2 in the Supplementary material. Nucleotide sequences for pCLP, pSLA-2, and *S. avermililis* chromosomes were obtained from RefSeq/GenBank accession numbers NC_004963.1, NC_024971.1, and BA000030.4, respectively.

Given that automated assembly pipelines may not provide a complete sequence for linear replicons (Table 1, Fig. 2a), we used 2 different approaches to refine the assemblies. The first involved retrieving unmapped paired-end reads, merging overlapping reads, and attaching both ends of the initial linear contig as overhangs, yielding a complete sequence of 348,162-bp linear replicon for OCU901s_S2_2s, with poly-C nucleotide sequences at both ends (Fig. 2b).

This linear replicon was named pS2b. To validate the assembled terminal regions, we performed restriction fragment length analysis on the proteinase K-treated genomic DNA of OCU901s_S2_2s, which confirmed that the poly-C nucleotides (Fig. 2b) represented the authentic terminal sequence of this replicon (Figure S1). A large portion of linear contig of strain BA018 exhibited similarity to pS2b. Therefore, the same refinement approach was applied to BA018 linear replicon, yielding a 366,138-bp complete sequence designated as pBA018a.

Linear contigs of PPE_S58, BA059, and BA705 also showed similarities to pS2b. For strains PPE_S58, BA059, and BA705, we employed a different assembly refinement approach that combined hybrid assembly, short-reads-only assembly, and, when necessary, the raw reads (see Materials and methods). This approach generated complete linear replicons larger than 300 kb with 5′ poly-C nucleotide ends: 456,679 bp for pPPE_S58a, 344,653 bp for pBA059a, and 308,290 bp for pBA705a (Fig. 2a, Figure S2a). Each replicon harbours approximately 415-bp terminal inverted repeats (TIRs) at both termini, with small insertions or deletions (1 to 23 bp) observed within the repeats. Linear chromosomes and plasmids in *Streptomyces* typically harbour 5 to 8 short inverted repeats, which can form stem loops in the single-stranded form of lagging-strand template in their telomeric regions.^53^ Similarly, the 23-kb *Mycobacterium celatum* linear plasmid pCLP harbours 3 stem-loop motifs at its telomeric region and poly-C nucleotides at the 5′ ends (Figure S2b).^54^ In our study, 6 to 7 stem-loop motifs were identified within the single-stranded form of 300 nt 3′ ends of 415-bp TIRs (designated I–VII in Fig. 2b and Figure S2a). Therefore, pS2b-type linear replicons have the sequence features of invertrons.^55^

**Fig. 3.**
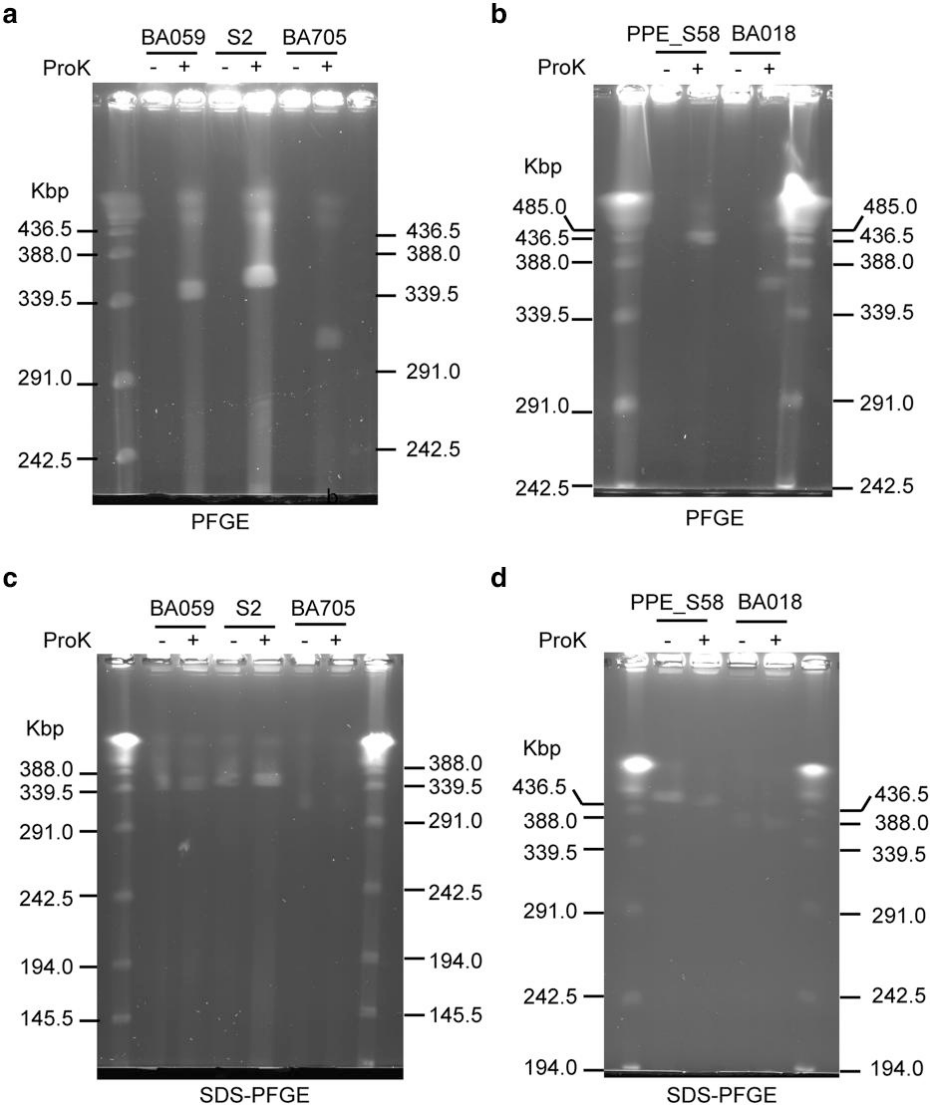
PFGE and SDS-PFGE of 5 *M. avium* strains. (a) PFGE profiles of strains BA059, OCU901s_S2_2s, and BA705. (b) PFGE profiles of strains PPE_S58 and BA018. (c) SDS-PFGE profiles of strains BA059, OCU901s_S2_2s, and BA705. (d) SDS-PFGE profiles of strains PPE_S58 and BA018. A heat-treated Lambda ladder (Bio-Rad) was used as the molecular size marker.

To further validate the assembly results and the invertron characteristics of the linear replicons, we conducted PFGE. In PFGE, only DNA molecules with a linear topology can migrate from the lysed cells embedded in agarose plugs.^56^ The termini of linear replicons can be linked to the terminal protein^57,58^ or form covalently closed hairpin structures with complementary strands.^59–61^ If a terminal protein is attached to the ends of linear replicons, such as invertrons, the protein-linear replicon complex cannot migrate from the lysed cells at plug positions during PFGE unless the protein is denatured using SDS or cleaved by proteases.^54,56,57,62,63^ As shown in Fig. 3a and b, a DNA molecule of a specific size migrated to the gel only when the cell-embedded plug was treated with proteinase K, and its size in PFGE gels corresponded to the *in silico* assembly results. When SDS was added to the electrophoresis buffer, the same DNA molecule migrated, regardless of the proteinase K treatment (Fig. 3c and d). This indicates that the linear replicons identified *in silico* are extrachromosomal elements whose termini are covalently linked to as-yet-unknown terminal proteins, as is the case with other invertrons.

### 3.3. Linear replicons are linear plasmids

Previous studies have reported an association between tRNA array units and bacteriophages.^24,26^ The copy numbers of the 5 linear replicons in the cell were estimated to be approximately 1.0 to 1.5 copies per chromosome across strains based on short-read coverage (Table 1). Therefore, linear replicons have a copy number control function: one requirement to be called plasmid. This raises the question: Are these linear replicons phage-plasmids or bona fide plasmids? A total of 324 protein-coding sequences (gene families) were conserved across all 5 linear replicons using PIRATE- and DFAST-based annotation.^46,47^  Table S2 provides notable gene families conserved across 5 linear replicons.

The notable core gene families include (i) genes encoding type VII secretion system (EccA, EccB, EccC, EccD, EccE, MycP), the type IV secretion system (VirB4, VirB8, also known as TcpC), and MinD-type ATPase (COG0455), which together form a gene cluster; (ii) a gene encoding a ParA-type AAA family ATPase (COG1192); (iii) 3 types of peptidase genes—TIGR04338 family metallohydrolase, Rv3717 family N-acetylmuramoyl-L-alanine amidase (COG0860), and NlpC-type cell wall-associated hydrolase (COG0791); and (iv) 4 types of helicase genes—DnaB-type replicative DNA helicase (COG0305), UvrD-type superfamily I DNA or RNA helicase (COG0210), DNA2-type superfamily I DNA or RNA helicase (COG1112), and superfamily II DNA or RNA helicase (COG1061) (Fig. 2). We were unable to identify genes encoding replication initiation proteins or the replication origin *oriV* of this linear replicon through similarity searches.

BLASTP searches of pS2b-encoded protein sequences against the Actinobacteriophage database^64^ did not yield any significant matches at an E-value threshold of <0.1. The best-characterized mycobacterial conjugative plasmid group, the pRAW-related plasmids,^65^ which include the *M. avium* plasmid pMAH135,^22^ encode a hybrid type VII and type IV secretion system (T7/4SS) and a relaxase protein often annotated as TraA.^65^ All circular replicons other than chromosomes in the 5 sequenced strains encode TraA relaxase homologues, such as product of locus_tag OCU901_53400 of pS2a. However, TraA relaxase homologues were not identified in any of the 5 linear replicons. The *Streptomyces* circular plasmid pSVH1 and the integrative and conjugative element pSAM2 are known to transfer among *Streptomyces* cells in double-stranded DNA form mediated by an FtsK homologue, without the involvement of T4SS or relaxase.^66,67^ The EccC protein contains an FtsK domain. Thus, the absence of relaxase homologues in the pS2b-type replicon does not necessarily indicate a lack of transfer activity in this genus. Rather, it implies that the transfer of pS2b-type linear replicon does not involve rolling-circle-like replication during horizontal transfer, a mechanism often used by circular plasmids that use T4SS. The intracellular copy number, genetic similarity to pRAW, and the presence of pS2b-related segments in multiple *M. avium* subpopulations (discussed below) collectively suggest that the 5 linear replicons are low-copy-number conjugative plasmids.

### 3.4. ESX-P locus of pS2b-type linear plasmids

**Fig. 4.**
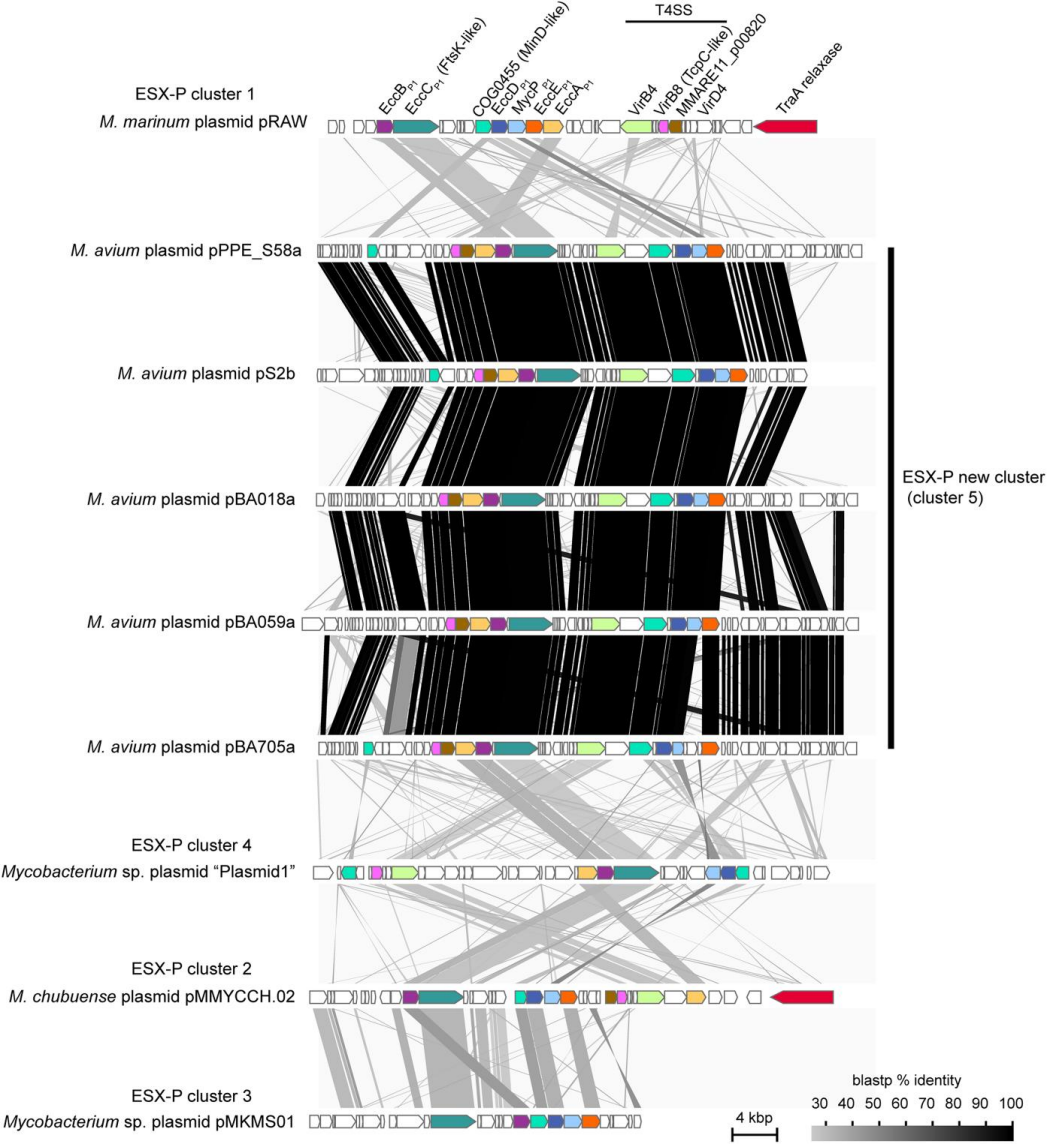
Gene synteny comparison of ESX-P loci. Orthologous genes are represented in matching colours. ESX-P cluster numbers follow the classification system proposed by Dumas *et al*..^71^ Sequences were obtained from the following GenBank/RefSeq accession numbers: HG917973.1 (pRAW), NC_021278.1 (plasmid1), AP041819 (pS2b), AP038890.1 (pPPE_S58a), AP038886.1 (pBA705a), AP038880.1 (pBA059a), AP038876.1 (pBA018a), CP003055.1 (pMMYCCH.02), and NC_008703.1 (pMKMS01). The *traA* homologue is absent in ESX-P cluster 5. pMKMS01 carries a *traA* homologue outside the region shown in the panel.

To date, only a few studies have reported the presence of linear plasmids in members of the family Mycobacteriaceae.^54,68,69^ pCLP from *Mycobacterium celatum* is the only mycobacterial linear plasmid to date that has formally been demonstrated to be fully sequenced from end to end using a cloning approach.^54,70^ pCLP is similar to pS2b-type plasmids because it harbours poly-C nucleotides at both ends and terminal sequences that form imperfect inverted repeats (Fig. 2a and Figure S2b).^54^ However, unlike the pS2b-type plasmids, pCLP lacks the T7/4SS locus.^70^ Plasmid-borne T7SS locus was previously termed ESX-P.^71^ The overall synteny of T7/4SS genes in the pS2b-type plasmids is shown with ESX-P cluster 1, cluster 2, cluster 3, and cluster 4 in Fig. 4. Notably, in contrast to the ESX-P1 locus found in pRAW-related circular plasmids, *eccA* (light orange in Fig. 4) is positioned upstream of *eccB* in pS2b-type linear plasmids. A T4SS gene, *virB4* homologue, is located between T7SS genes *eccD* and *eccC* in pS2b-type plasmids, whereas in pRAW, *virB4* homologue forms a cluster with another T4SS gene, *virB8* homologue.

A gene cluster containing *virB8* homologue and a hypothetical gene (magenta and dark brown in Fig. 4) in pRAW is 8.7 kb away from the T7SS gene *eccA* on pRAW, whereas it is positioned adjacent to *eccA* in pS2b-type plasmids. The *virD4* and *traA* homologues present in pRAW are absent from pS2b. Furthermore, the sequence identity of Ecc proteins between the pS2b homologue and the homologues from other reported plasmids was approximately 30% (Fig. 4). These observations indicate that the ESX locus found in pS2b-type linear plasmids constitutes a previously unclassified T7/4SS locus, different from the 10 T7SS loci identified in multiple *Mycobacterium* strains (ESX-1 to ESX-5, ESX-4bis, and ESX-P1–ESX-P4).^71^

**Fig. 5.**
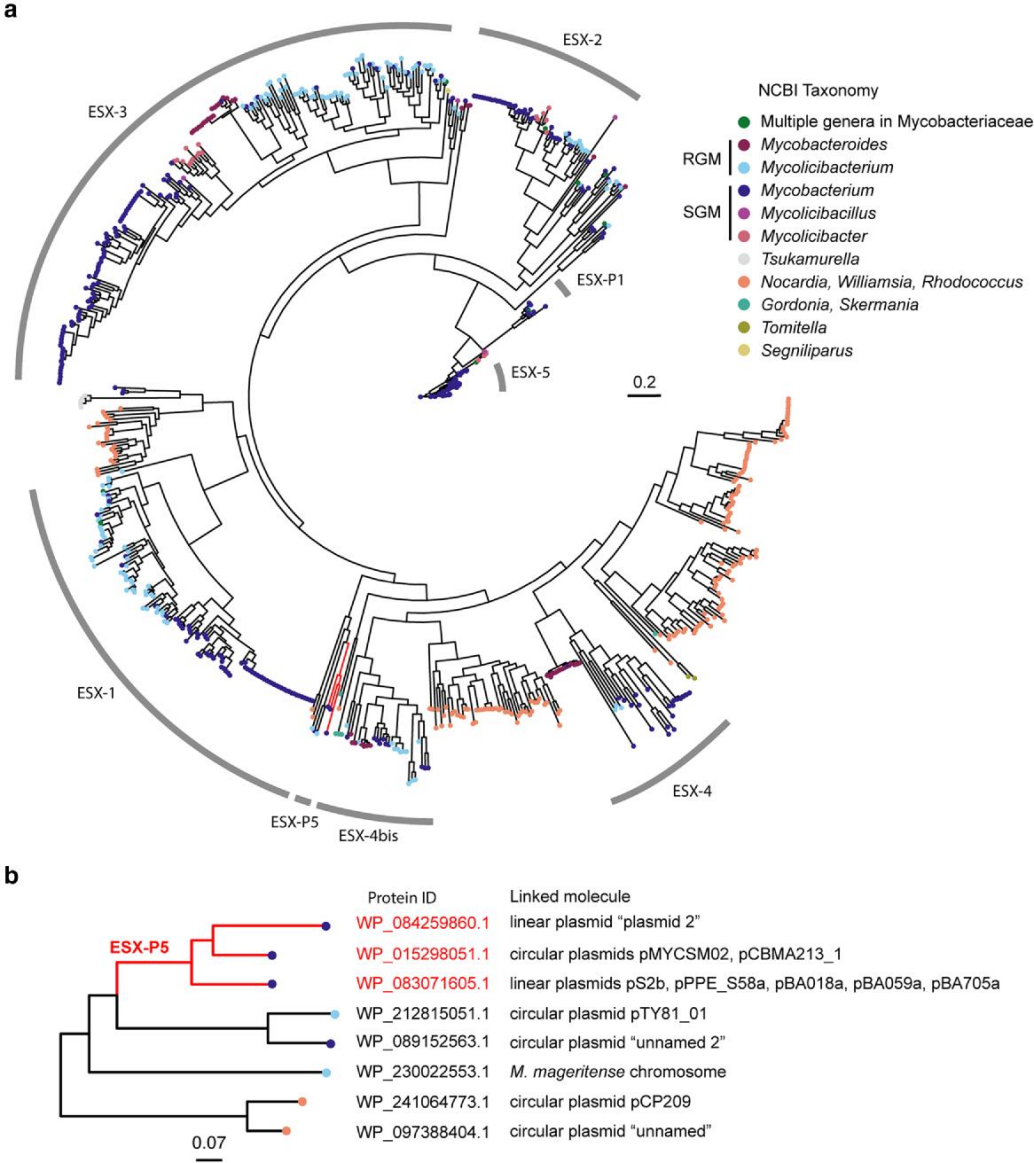
(a) Phylogenetic tree of the T7SS loci in the order Mycobacteriales based on EccB protein sequence alignment. Tip colours indicate the taxonomy of the DNA host. The consensus tree was generated using 10,000 bootstrap replicates under the nonreversible model implemented in IQ-TREE 2.^39^ ESX-5 is absent from the genomes of rapidly growing mycobacteria.^71^ A clade of plasmids sharing gene synteny with pS2b, designated ESX-P cluster 5 (ESX-P5), is highlighted in red. (b) A subset tree containing ESX-P5. Source protein ID and the associated molecule names were indicated by the tips. The highlighted branches are the same as shown in panel (a).

To explore the evolutionary origin of the pS2b-type linear plasmids, sequences of T7SS gene clusters from 1,613 complete genomes of the order Mycobacteriales (RefSeq database as of 1 November 2024) were retrieved and analysed phylogenetically, including those from pS2b plasmids. Figure 5a shows the phylogenetic tree of the T7SS locus based on EccB alignments. According to the EccB phylogenetic tree, the ESX-P locus of the linear plasmids shares its most recent common ancestor with a common ancestor of ESX locus of a hypothetically linear plasmid from *M. intracellulare* subsp. *chimaera* (protein ID: WP_084259860.1 in the subset tree in Fig. 5b) and a circular plasmid pMYCSM02 from *Mycobacterium* sp. (WP_015298051.1). The ancestor of these ESX loci then merge with a common ancestor of ESX loci from a circular plasmid from *M. intracellulare* subsp. *chimaera* (WP_089152563.1) and a circular plasmid from *Mycolicibacterium* sp. (WP_212815051.1) (light blue tips in Fig. 5b). The common ancestor of these ESX loci merges with an ESX locus of *Mycolicibacterium mageritense* chromosome (WP_230022553.1), then merges with the ancestor of T7SS loci of 2 circular plasmids from the genus *Rhodococcus* (orange tips in Fig. 5b) before ultimately merging with the chromosomal T7SS loci of *Nocardia*, mycobacterial ESX-4, and ESX-4bis (Fig. 5a). This pattern suggests that the ESX-P locus of pS2b-type linear plasmids likely originated from horizontally transferable elements within Mycobacteriales rather than from the ESX loci present on the *M. avium* chromosome.

Gene synteny within the ESX-P locus of pS2b is conserved across multiple plasmids (highlighted in red branches in Fig. 5b), with a reduced protein sequence identity. These includes the aforementioned plasmids from *M*. *intracellulare* subsp. *chimaera* (62 to 86% identity) and a circular plasmid pCBMA213_1 from *Mycolicibacterium* sp. (36 to 75% identity). Thus, the new ESX-P locus of the pS2b-type linear plasmids has been designated as the novel ESX-P “cluster 5” (hereafter ESX-P5).

### 3.5. Gene content similarity of pS2b-type linear plasmids with other NTM-harbouring plasmids

To gain further insight into the evolutionary relationships between pS2b-type linear plasmids and other plasmids identified in NTM, the gene content of pS2b-type linear plasmids was compared with that of 309 NTM plasmids submitted to RefSeq. The number of gene families identified per NTM plasmid ranged from 3 to 740, with a median of 36. Conversely, the number of gene families identified in the pS2b-type linear plasmid genomes ranged from 342 (pBA705a) to 463 (pPPE_S58a), constituting the second largest genome group in NTM plasmids (Table S3); the largest plasmid was an 864-kb putative linear plasmid from *Mycobacterium branderi* (Table S3). The tRNA array units were detected in both circular and putative linear plasmids but only in large plasmids (>200 kb) (Figure S3, Table S3), suggesting that the presence of a tRNA array unit is a genetic feature of large NTM plasmids. The aforementioned relaxase homologue was prevalent among the putative circular NTM plasmids; according to DFAST annotations, 193 of the 235 circular plasmids contained relaxase genes. Conversely, it was rare among the putative linear plasmids, with only 32 of 78 possessing a relaxase gene (Table S3). Although the topology information reported in GenBank does not reflect experimentally validated topology, the distribution of relaxase biased towards circular plasmid (odds ratio = 6.4, *P* = 2.8 × 10^−11^ in 1-sided Fisher’s exact test) suggests that the absence of relaxase may serve as an indicator of linear NTM plasmids.

Hierarchical clustering based on pairwise gene contents dissimilarities revealed that the 5 pS2b-type linear plasmids to some extent share gene families with a 437-kb putative linear plasmid (NZ_CP012886.2), a 322-kb circular plasmid (NZ_CP084587.1) and a 324-kb putative linear plasmid (NZ_LT703506.1), all from *M. intracellulare* subsp. *chimaera* (Figure S4a). The homologous genes shared among these 3 plasmids are clustered primarily in a 66-kb region of pS2b, which includes the ESX-P5 locus and a tRNA array unit. Additionally, pS2b and the 437-kb plasmid shared a 52-kb region (Figure S4b, bottom left). These findings suggest that the similarity between pS2b-type linear plasmids and *M. intracellulare* plasmids is limited to specific regions, with most plasmid genomes showing distinct ancestry.

Next, we specifically investigated whether any genes were shared between the pS2b-type linear plasmids and the reference mycobacterial linear plasmid pCLP.^54,70^ No shared gene families were identified except for the ParA-type AAA family ATPase and an IS*607* family-associated transposase, as revealed by pangenome analysis using a threshold of >50% identity. A further PHMMER search identified total 7 hypothetical proteins from pCLP with domain-level matches (E-value < 0.01) to 2 hypothetical proteins (locus_tags OCU901_49590, OCU901_50340) in pS2b. Although these proteins may be involved in maintaining linear plasmids, their functions cannot be inferred from currently available data.

Collectively, the gene composition analysis highlights the uniqueness of *M. avium* linear plasmids among NTM plasmids.

### 3.6. Characteristic features of pS2b-type linear plasmids among *M. avium* replicons

**Fig. 6.**
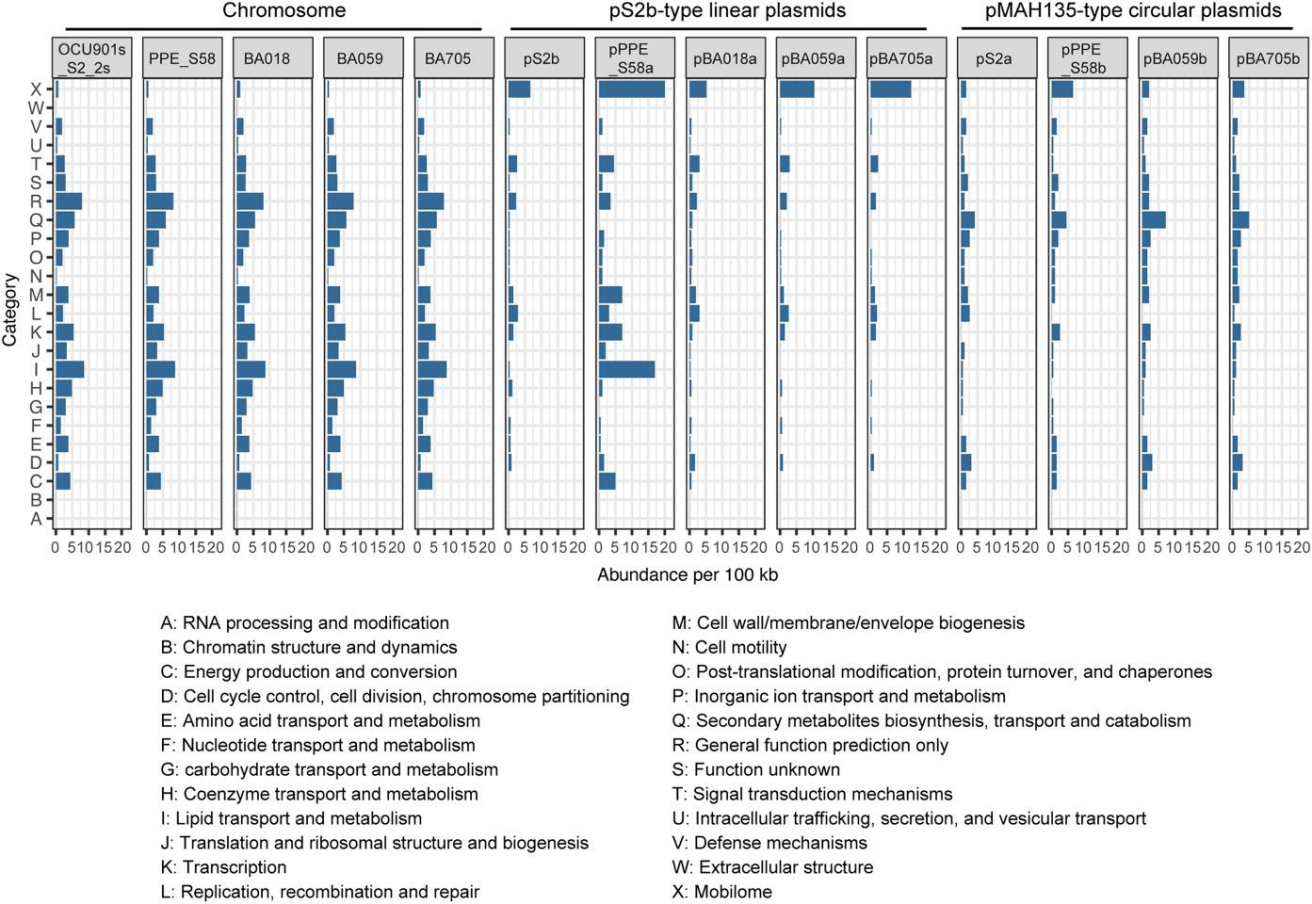
Abundance of functional categories based on COG hits. The abundance of each category was normalized to the size of the respective replicons.

To understand the biological functions associated with pS2b-type linear plasmids, the abundance of function categories associated with COG hits was compared among 3 major replicon types found in 5 *M. avium* strains sequenced in this study: chromosome, pS2b-type linear plasmid, and pMAH135-type circular plasmid. The proportion of coding sequences with COG hits was generally low in pS2b-type linear plasmids (66 of 418 proteins from pS2b showed hits) compared with pMAH135-type circular plasmids (57 of 162 proteins from pS2a showed hits). In pMAH135-type plasmids, categories Q and P were abundant (Fig. 6), reflecting the presence of multiple genes involved in nonribosomal peptide synthesis (COG1020) and metal transporter genes (COG2217 and COG0798). In linear plasmids, category X, mostly linked to transposases, was the most abundant function category, exceeding that of the other 2 major replicon types. Therefore, the function category frequency pattern clearly differs among 3 types of major replicons of *M. avium*.

Consistent with this functional category analysis, IS density was highest in pS2b-type linear plasmids (Figure S5): 2.6 to 4.6 copies per 100 kb in 5 linear plasmids, contrasting with 0.41 to 0.88 copies per 100 kb in chromosomes and 1.0 to 2.0 copies per 100 kb in 4 pMAH135-type circular plasmids. In *Streptomyces* genomes, IS elements accumulate at the terminus regions of linear chromosomes.^20,72^ By contrast, the locations of IS elements within pS2b-type linear plasmids varied among plasmids, and no common insertion-site bias was observed. Currently, factors other than gene nonessentiality promoting IS enrichment in pS2b-type linear plasmids remain unknown.

### 3.7. 153-kb island in pPPE_S58a

**Fig. 7.**
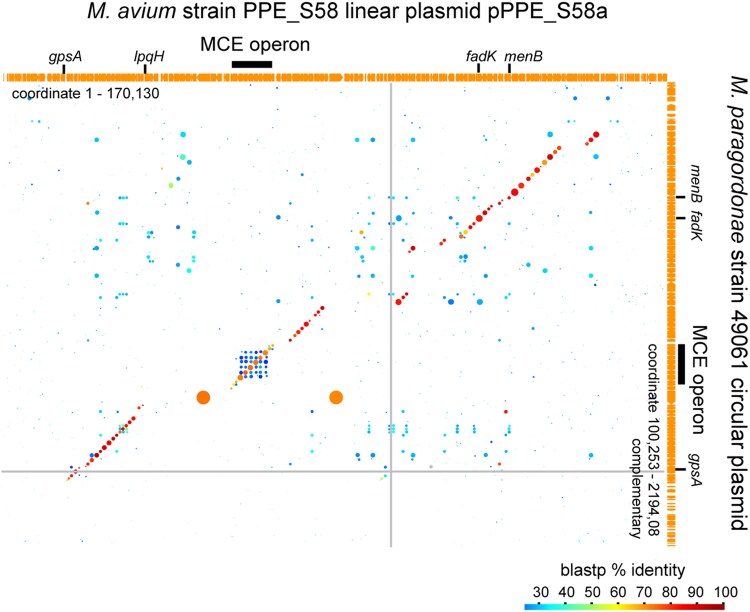
A synteny block is shared between pPPE_S58a and a plasmid from *M. paragordonae* strain 49061. BLASTP hits are shown as circles in the 2-dimensional plots. The 2 large circles represent BLAST hits between the paralogs of the serine/threonine-protein kinase genes. Sequence was obtained from RefSeq accession number NZ_CP025547.1. A similar synteny block is also present in the draft genome of *M. kiyosense* (NZ_BRZK01000003.1).

COG hits associated with functional categories I, K, and M were exceptionally abundant in pPPE_S58a among the 5 linear plasmids (Fig. 6). This reflects the presence of a unique 153-kb island (coordinates 5,345 to 158,500) associated with lipid transport and metabolism, as highlighted in Fig. 7. Notable genes in the 153 kb region include (i) an MCE operon, putatively involved in cell wall lipid homeostasis (category M)^73^; (ii) 2 paralogous genes encoding serine/threonine-protein kinase (category T); (iii) a *lpqH* homologue encoding a small lipoprotein that may induce TLR2-dependent apoptosis^74–76^; (iv) a *menB* homologue encoding naphthoate synthase for vitamin K2 biosynthesis (category H)^77^; and (v) several genes involved in lipid biosynthesis or degradation, including *fadK* (category I), *fabG* (category I), and regulator gene *fadR* (category K). An equivalent island sequence, excluding the *lpqH* homologue region, was present in a circular plasmid from *Mycobacterium paragordonae* and a contig from *Mycobacterium kiyosense*^78^—both belonging to the *Mycobacterium gordonae* complex (Fig. 7) but absent from *M. avium* chromosomes. These 2 plasmid-like sequences showed no similarity to the other regions of pPPE_S58a. Therefore, the majority of the synteny block in the 153-kb island of pPPE_S58a may have been established in an NTM plasmid in the past and disseminated among NTM plasmids through translocation.

### 3.8. Distribution of pS2b-type linear plasmids in *M. avium* subsp. *hominissuis*

**Fig. 8.**
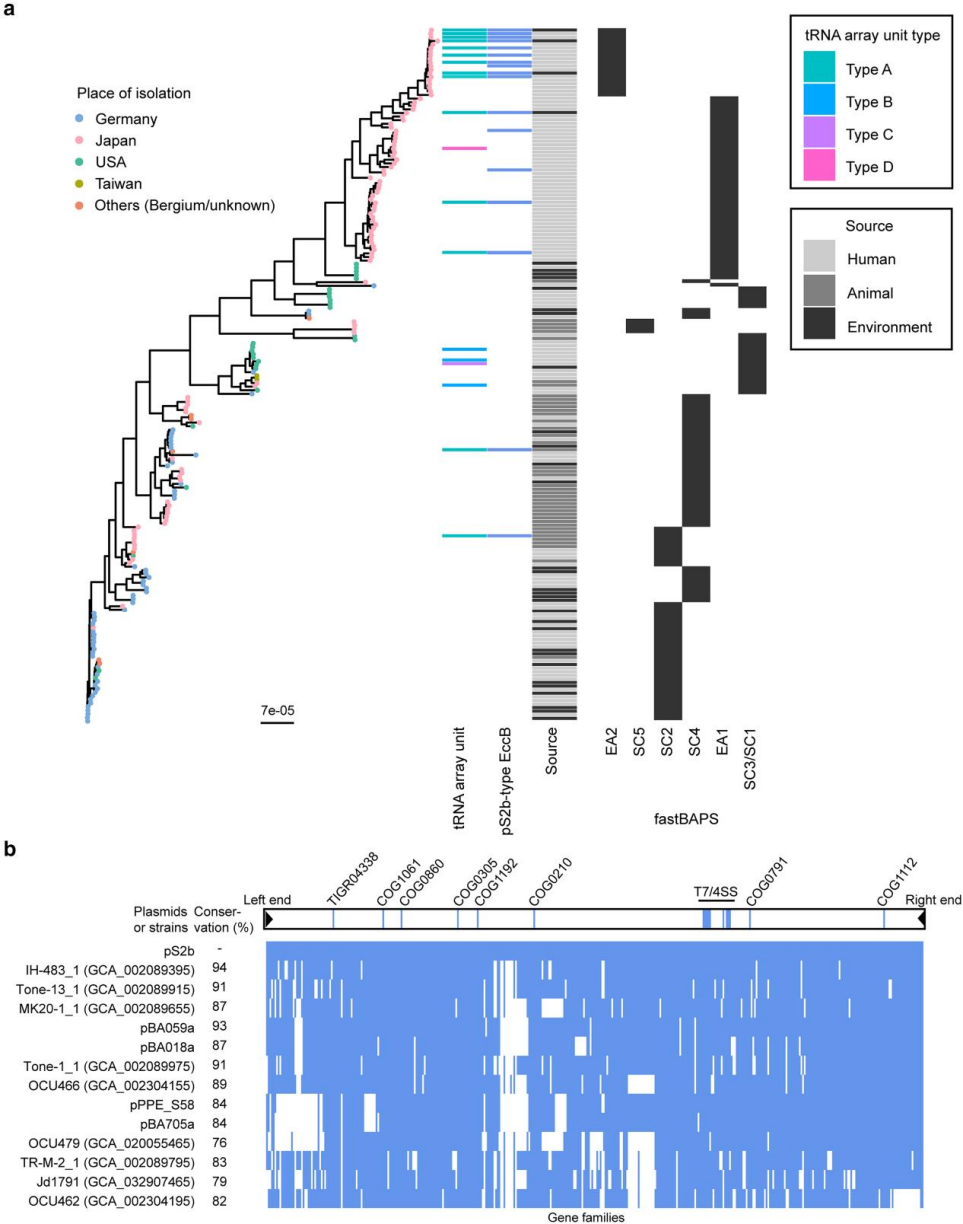
(a) Distribution of pS2b-type linear plasmids in *M. avium* subsp. *hominissuis*. Left: maximum-likelihood phylogenetic tree of 193 strains based on chromosomal sequence alignments. The scale bar indicates the substitution rate. The tree was inferred using IQ-TREE 2 with the GTR model^39^ and corrected with ClonalFrameML.^45^ Tip colours indicate the place of isolation. Centre: Presence/absence pattern for the tRNA array unit, pS2b-type EccB, and isolation source. Right: Population structure inferred using fastBAPS.^79^ Subpopulation names (EA1, EA2, SC2, SC4, SC3/SC1, SC5) are consistent with nomenclatures used to describe BAPS3 sequence clusters or fastGEAR lineages in previous studies.^5,6,80^ (b) Conservation of gene families detected in pS2b in other 4 linear plasmids and 9 draft genomes. Gene families identified by PIRATE are sorted according to their locations on pS2b. Locations of notable core genes with COG annotation were indicated over the binary matrix of gene families.

The absence of pS2b-type linear plasmids among sequences annotated as “plasmid” in GenBank suggests a geographically biased rather than niche-specific distribution, as we identified these linear plasmids in both bathroom and human isolates. To test this hypothesis, we searched for segments characteristic of pS2b-type linear plasmids (*eccA*, *eccB*, and the tRNA array unit) in the draft genome sequences of *M. avium* subsp. *hominissuis* in GenBank. After merging 5 complete genomes, 17 of 193 genomes (8.8%) encoded both *eccA* and *eccB* proteins with high similarity to pS2b homologues (BLASTN identity > 98%). Using the current dataset, the population was divided into 6 subpopulations through Bayesian hierarchical clustering (fastBAPS^79^) based on the composition of chromosomal SNPs: MahEastAsia1 (EA1), MahEastAsia2 (EA2), SC2, SC4, SC5, and a merged group comprising the previously designated SC1 and SC3^6,80^ (Fig. 8a). The 17 strains were assigned to EA1 (*n* = 5), EA2 (*n* = 10), SC2 (*n* = 1), or SC4 (*n* = 1) subpopulations. Strains OCU901s_S2_2s, PPE_58, BA059, and BA705 belonged to EA2, whereas BA018 belonged to EA1. At the draft genome assembly level, 14 of these 17 genomes harboured a tRNA array unit. Pangenome analysis of the 5 pS2b-type linear plasmids and 9 draft genomes harbouring both the *eccB* gene and the Type A tRNA array unit revealed that 72% to 94% of gene families present in pS2b were conserved in the draft genomes (Fig. 8b). Conserved genes include coding sequences of the left and right terminal regions (locus_tags: PPES58_48270–PPES58_48280 for the left end and PPES58_53510–PPES58_53520 for the right end in pPPE_S58a). Thus, 9 additional strains in the database likely harbour a pS2b-type linear plasmid. When the pS2b genome was divided into 3 sectors, the right sector—which includes the T7/4SS locus—was more highly conserved than the other sectors. Conversely, several synteny blocks in the central and left sectors were absent from genomes other than pS2b, suggesting that these regions of pS2b are dispensable for the maintenance of the linear plasmids.

All 14 strains harbouring the Type A array unit also harboured ESX-P cluster 5 (hereafter ESX-P5), suggesting the coevolution of these 2 genomic segments. We speculate that the tRNA array unit was unidentified in the 3 *M. avium* strains, despite the presence of ESX-P5 (Fig. 8a), due to incomplete genome assemblies of short reads for the tRNA array unit region. Interestingly, a plasmid from *M. intracellulare* MC045 harboured the Type D tRNA array unit (Fig. 1a), and both the array unit and its flanking sequences were identified in the draft genome of *M. avium* DH-6 (GCA_002089175.1). This suggests that *M. avium* DH-6 may harbour a plasmid originating from *M. intracellulare* subsp. *chimaera*.

The presence of pS2b-related segments was biased towards specific genetic subpopulations: 10 of 19 strains in EA2, 5 of 52 strains in EA1, 1 of 44 strains in SC2, and 1 of 51 strains in SC4. The most plausible explanation for this biased distribution is that pS2b-type plasmids originated in EA2 and were subsequently transmitted to other subpopulations, rather than having been present in a common ancestor of each subpopulation and subsequently lost through segregation at many terminal branches of the phylogenetic tree (Fig. 8a). It is therefore more parsimonious to interpret pS2b-type plasmids as transferable rather than nontransferable elements. Among the 14 *M. avium* strains that likely harbour a pS2b-type linear plasmid, 13 strains were isolated in Japan (excluding one dog isolate for which location data are not publicly available), regardless of their genetic subpopulation classification. This supports the hypothesis that pS2b-type linear plasmids were first established in *M. avium* subsp. *hominissuis* or another NTM in Asia and have not yet spread globally to a detectable extent.

## 4. Conclusion

The established hybrid assembly technique does not always yield circular contigs for *M. avium* genomes. This study formally examined whether linear contigs are plasmids. Our findings indicate that *M. avium* harbours giant linear plasmids that carry a tRNA array unit and that the terminal region information from these linear plasmids is preserved in Illumina shotgun sequencing libraries. The novel identification of giant linear plasmids in *M. avium* broadens our understanding of the genomic architecture of NTM and supports a more accurate interpretation of genome assembly data. The 5 reference genomes established in this study provide a valuable foundation for future data analysis designs that account for plasmids in NTM sequencing projects.

## Supplementary Material

dsaf039_Supplementary_Data

## Data Availability

Raw sequencing reads generated in this study are available under BioProject PRJDB19714. Nucleotide sequences of the 5 *M. avium* strains have been deposited under the following GenBank/DDBJ/EMBL accession numbers: OCU901s_S2_2s chromosome, AP041818; pS2a, AP041820; pS2b, AP041819; pS2c, AP041822; cS2d, AP041821; BA018 chromosome, AP038875; pBA018a, AP038876; pBA018b, AP038877; pBA018c, AP038878; BA059 chromosome, AP038879; pBA059a, AP038880; pBA059b, AP038881; pBA059c, AP038882; pBA059d, AP038883; pBA059e, AP038884; BA705 chromosome, AP038885; pBA705a, AP038886; pBA705b, AP038887; pBA705c, AP038888; pBA705d, AP038889; PPE_S58 chromosome, pPPE_S58a, AP038890; pPPE_S58b, AP038891; pPPE_S58c, AP038892; pPPE_S58d, AP038893. Datasets used are available from the Zenodo repository (URLs: https://doi.org/10.5281/zenodo.15823864; https://doi.org/10.5281/zenodo.15822127; https://doi.org/10.5281/zenodo.15824003).^81–83^ Phylogenetic trees with tip labels, DFAST annotations, and scripts used in this study are available from the Figshare repository (URL: http//doi.org/10.6084/m9.figshare.29485367).^84^
