# Supplementary material for: Giant linear plasmids in Mycobacterium avium harbour a tRNA array unit
Source: DNA Res. 2026 Jan 3;33(1):dsaf039. doi: 10.1093/dnares/dsaf039 (PMC12803027; doi:10.1093/dnares/dsaf039)
Supplement: dsaf039_Supplementary_Data [file dsaf039_supplementary_data.zip › Fig S1.docx]

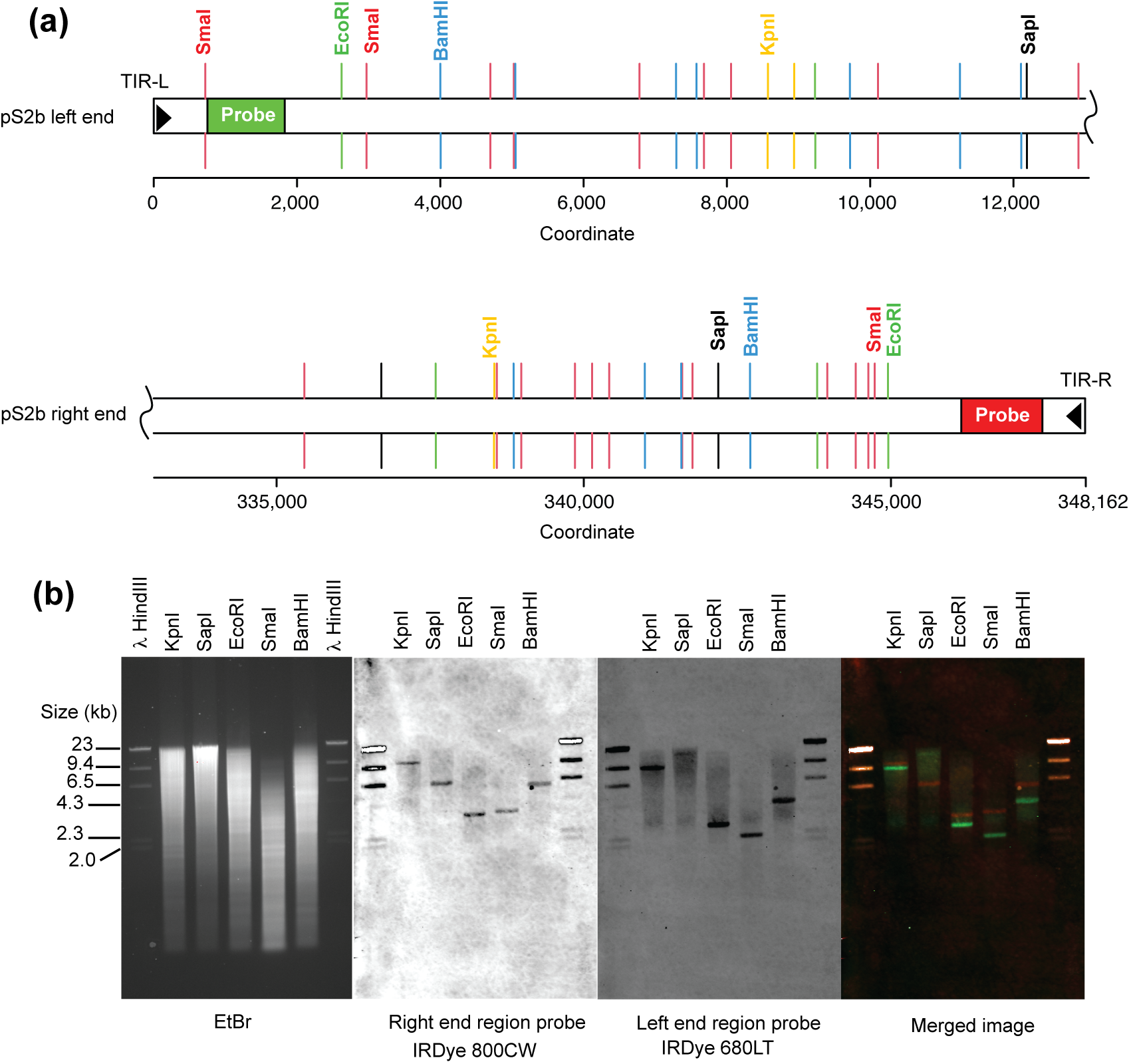


# Fig. S1. Physical map of the pS2b terminal regions. (a) Restriction map of the terminal regions of pS2b. Regions used as probes for Southern hybridization are shown in green or red. (b) Southern hybridization results. DNA visualized by ethidium bromide staining (left) was transferred to a BioDyne membrane and hybridized simultaneously with probes targeting the left and right terminal regions. The merged signals from the two probes are shown in the right panel: green indicates the left-end probe, and red indicates the right-end probe.
