# Supplementary material for: Giant linear plasmids in Mycobacterium avium harbour a tRNA array unit
Source: DNA Res. 2026 Jan 3;33(1):dsaf039. doi: 10.1093/dnares/dsaf039 (PMC12803027; doi:10.1093/dnares/dsaf039)
Supplement: dsaf039_Supplementary_Data [file dsaf039_supplementary_data.zip › Fig S2.docx]

**(a)**

IV

II

III

I

. . . . . . . . . .

pS2b_L 1 -ccccccccgcggcttcgccgcacagcggagaccgccggcgcaagcgccgacgacgcacgccggcctccggccggctacccccgttgcgggt-caattat 98

pS2b_R(rc) 1 cccccccccgcggcttcgccgca--gcggagaccgccggcgcaagcgccgacgacgcacgccggcctccggccggctacccccgttgcggggtcaattat 98

pPPE_S58a_L 1 cccccccccgcggcttcgccgca-----------------------gccgacgacgcacgccggcctccggccggctaccaccgctgcgggt-caattat 76

pPPE_S58a_R(rc) 1 -ccccccccgcggcttcgccgca--gccgcgaacgcggccgccagaggcgacgacgcacgccggcctccggccggctacccccgttgcgggt-caattat 96

pBA705a_L 1 cccccccctgcggcttcgccgca--gcggagaccgccggcgcaagcgccgacgacgcacgccggcctccggccggctaccaccgctgcgggt-caattat 97

pBA705a_R(rc) 1 cccccccccgcggcttcgccgca--gcggagaccgccggcgcaagcgccgacgacgcacgccggcctccggccggctacccccgttgcgggt-caattat 97

pBA059a_L 1 -ccccccccgcggcttcgccgca-agcggagaccgccggcgcaagcgccgacgacgcacgccggcctccggccggctaccccccttgcgggt-caattat 97

pBA059a_R(rc) 1 -ccccccccgcggcttcgccgca-agcggagaccgccggcgcaagcgccgacgacgcacgccggcctccggccggctacccccgttgcgggt-caattat 97

pBA018a_L 1 -ccccccccgcggcttcgccgca--gcggagaccgccggcgcaagcgccgacgacgcacgccggcctccggccggctacccccgttgcgggt-caattat 96

pBA018a_R(rc) 1 -ccccccccgcggcttcgccgca--gcggagaccgccggcgcaagcgccgacgacgcacgccggcctccggccggctacccccgttgcgggt-caattat 96

consensus cCCCCCCCCGCGGCTTCGCCGCAcaGCGGaGAcCgCcGGCGCaAGcGcCGACGACGCACGCCGGCCTCCGGCCGGCTACCcCCGtTGCgGGttCAATTAT

VI

V

. . . . . . . . . .

pS2b_L 99 gttgtgcgcgttgtcttttcgatgtggtggatggttgcggtcgcggcttcgccgcgaccgggttacgtcgctgtatgttgttgtgttgatttgggtttcg 198

pS2b_R(rc) 99 gttgtgcgcgttgtcttttcgatgtggtggatggttgcggtctcggcttcgccgtgaccgggttacgtggctgtgtgttgttgtgttgcttggggttttg 198

pPPE_S58a_L 77 gttgtgcgcgttgtcttttcgatgtggtggatggttgcggttgcggcttcgccgcgaccgggttacgtggctgtatgttgttgtgttgatttgggtttcg 176

pPPE_S58a_R(rc) 97 gttgtgcgcgttgtcttttcgatgtggtgg------gcggtcgcggcttcgccgcgaccgggttacgtggctgtgtgttgttgtgttgcttggggttttg 183

pBA705a_L 98 gttgtgcgcgttgtcttttcgatgtggtggatggttgcggttgcggcttcgccgcgaccgggttacgtggctgtatgttgttgtgttgatttgggtttcg 197

pBA705a_R(rc) 98 gttgtgcgcgttgtcttttcgatgtggtggatggttgcggttgcggcttcgccgcgaccgggttacgtggctgtgtgttgttgtgttgcttggggttttg 197

pBA059a_L. 98 gttttgcgcgttgtcttttagatgtggtggatgg----ggtcgcggcttcgccgcgaccgggttacgtcgctgtatgttgttgtgttgatttgggtttcg 193

pBA059a_R(rc) 98 gttgtgcgcgttgtcttttcgatgtggtggatggttgcggtcgcggcttcgccgcgaccgggttacgtggctgtgtgttgttgtgttgcttggggttttg 197

pBA018a_L 97 gttgtgcgcgttgtcttttcgatgtggtggatggttgcggtcgcggcttcgccgcgaccgggttacgtcgctgtatgttgttgtgttgatttgggtttcg 196

pBA018a_R(rc) 97 gttgtgcgcgttgtcttttcgatgtggtggatggttgcggtcgcggcttcgccgcgaccgggttacgtggctgtgtgttgttgtgttgcttggggttttg 196

consensus GTTGTGCGCGTTGTCTTTTcGATGTGGTGGatggttgCGGTcgCGGCTTCGCCGcGACCGGGTTACGTgGCTGTgTGTTGTTGTGTTGaTTgGGGTTTtG

VII

. . . . . . . . . .

pS2b_L 199 tgcgacccattcttcggcttcgcctccttgtcgaatatcgttgcattaccggttgtttcatgccagctcgcggctgcgccgcttactcca-ttgtatccc 297

pS2b_R(rc) 199 tgcgacccattcttcggcttcgcctccttgtcgaatatcgttgcgttatcggttgtttcatgccagctcgcggcttcgccgcttactccatttgtatccc 298

pPPE_S58a_L 177 tgcgacccattcttcggcttcgcctccttgtcgaatatcgttgcgttaccggttgtttcatgccagctcgcggcttcgccgcttactcca-ttgtatccc 275

pPPE_S58a_R(rc)184 tgcgacccattcttcggcttcgcctccttgtcgaatatcgttgcgttatcggttgtttcatgccagctcgcggcttcgccgcttactccatttgtatccc 283

pBA705a_L 198 tgcgacccattcttcggcttcgcctccttgtcgaatatcgttgcgttaccggttgtttcatgccagctcgcggcttcgccgcttactcca-ttgtatccc 296

pBA705a_R(rc) 198 tgcgacccattcttcggcttcgcctccttgtcgaatatcgttgcgttatcggttgtttcatgccagctcgcggcttcgccgcttactccatttgtatccc 297

pBA059a_L 194 tgcgacccattcttcggcttcgcctccttgtcgaatatcgttgcattaccggttgtttcatgccagctcgcggctgcgccgcttactcca-ttgtatccc 292

pBA059a_R(rc) 198 tgcgacccattcttcggcttcgcctccttgtcgaatatcgttgcgttatcggttgtttcatgccagctcgcggcttcgccgcttactccatttgtatccc 297

pBA018a_L 197 tgcgacccattcttcggcttcgcctccttgtcgaatatcgttgcattaccggttgtttcatgccagctcgcggctgcgccgcttactcca-ttgtatccc 295

pBA018a_R(rc) 197 tgcgacccattcttcggcttcgcctccttgtcgaatatcgttgcgttatcggttgtttcatgccagctcgcggcttcgccgcttactccatttgtatccc 296

consensus TGCGACCCATTCGGCGGCTTCGCCTCCTTGTCGAATATCGTTGCGTTAtCGGTTGTTTCATGCCAGCTCGCGGCTTCGCCGCTTACTCCAtTTGTATCCC

. . . . . . . . . .

pS2b_L 298 aatgcttttcgtactatcaccccatttcgactatggcgcaaggtctttcctcctttccggcgcggcagtacgggacagctgacgctgcccctcatacttt 397

pS2b_R(rc) 299 actgcttttcgtactatcaccccatttcgactatggcgcaaggtctttcctcctttccggcgcggcagtacgggacagctgacgcttgcccctcatactt 398

pPPE_S58a_L 276 aatgcttttcgtactatcaccccatttcgactatggcgcaaggtctttcctcctttccggcgcggcagtacgggacagctgacgc-tgcccctcatactt 374

pPPE_S58a_R(rc)284 actgcttttcgtactatcaccccatttcgactatagcgcaaggtctttcctcctttccggcgcggcagtacgggacagctgacgcttgcccctcatactt 383

pBA705a_L 297 aatgcttttcgtactatcaccccatttcgactatggcgcaaggtctttcctcctttccggcgcggcagtacgggacagctgacgc-tgcccctcatactt 395

pBA705a_R(rc) 298 actgcttttcgtactatcaccccatttcgactatagcgcaaggtctttcctcctttccggcgcggcagtacgggacagctgacgcttgcccctcatactt 397

pBA059a_L. 293 aatgcttttcgtactatcaccccatttcgactatggcgcaaggtctttcctcctttccggcgcggcagtacgggacagctgacgc-tgcccctcatactt 391

pBA059a_R(rc) 298 actgcttttcgtactatcaccccatttcgactatggcgcaaggtctttcctcctttccggcgcggcagtacgggacagctgacgcttgcccctcatactt 397

pBA018a_L 296 aatgcttttcgtactatcaccccatttcgactatggcgcaaggtctttcctcctttccggcgcggcagtacgggacagctgacgc-tgcccctcatactt 394

pBA018a_R(rc) 297 actgcttttcgtactatcaccccatttcgactatggcgcaaggtctttcctcctttccggcgcggcagtacgggacagctgacgcttgcccctcatactt 396

consensus AcTGCTTTTCGTACTATCACCCCATTTCGACTATGGCGCAAGGTCTTTCCTCCTTTCCGGCGCGGCAGTACGGGACAGCTGACGCttGCCCCTCATACTT

. . . . . . . . . .

pS2b_L 398 -caacgcttgccttcacaaccccaccacccaattgaggcgtcctgcttcccgatacctg---cgcacgtctgcgctctcaggtgcctttccgttgtct-- 491

pS2b_R(rc) 399 tcaatgcttgccttcgctcccctacccc----------cgtcct—ttgccgccaccggccccgctcgcttggtatgaggggcacaatccccatgctttcg 487

pPPE_S58a_L 375 tcaacgcttgccttcacaaccccaccacccaattgaggcgtcctgcttcccgatacctg---cgcacgtctgcgctctcaggtgcctttccgttgtct-- 469

pPPE_S58a_R(rc)384 tcaatgcttgccttcgctcccctacccc----------cgtcct—ttgccgccaccggccccgctcgcttggtatgaggggcacaatccccatgctttcg 472

pBA705a_L 396 tcaacgcttgccttcacaaccccaccacccaattgaggcgtcctgcttcccgatacctg---cgcacgtctgcgctctcaggtgcctttccgttgtct—- 490

pBA705_R(rc) 398 tcaatgcttgccttcgctcccctacccc----------cgtcct—ttgccgccaccggccccgctcgcttggtatgaggggcacaatccccatgctttcg 486

pBA059a_L 392 tcaacgcttgccttcacaaccccaccacccaattgaggcgtcctgcttcccgatacctg---cgcacgtctgcgctctcaggtgcctttccgttgtct—- 486

pBA059a_R(rc) 398 tcaatgcttgccttcgctcccctacccc----------cgtcct—ttgccgccaccggccccgctcgcttggtatgaggggcacaatccccatgctttc- 486

pBA018a_L 395 tcaacgcttgccttcacaaccccaccacccaattgaggcgtcctgcttcccgatacctg---cgcacgtctgcgctctcaggtgcctttccgttgtct—- 489

pBA018a_R(rc) 397 tcaatgcttgccttcgctcccctacccc----------cgtcct—ttgccgccaccggccccgctcgcttggtatgaggggcacaatccccatgctttcg 485

consensus TCAAtGCTTGCCTTC

**(b)**

. . . . . . . . . .

pCLP_L 1 ccccccccccccggcttcgccggagggaggacgccggcttacgccggctgacgcggccggcggctccgccgcctctctggcatggtggccggattgtgtt 100

pCLP_R(rc) 1 --ccccccccccggcttcgccgggtggagaggcagggct-acgccctgcaaactctgtcgcgcgcctgcggcgctctttgctgatcg--cgaattatgtt 95

consensus CCCCCCCCCCGGCTTCGCCGG GGAG GGCT ACGCC A GCG C GC GC T G CG ATT TGTT

**(c) (d)**


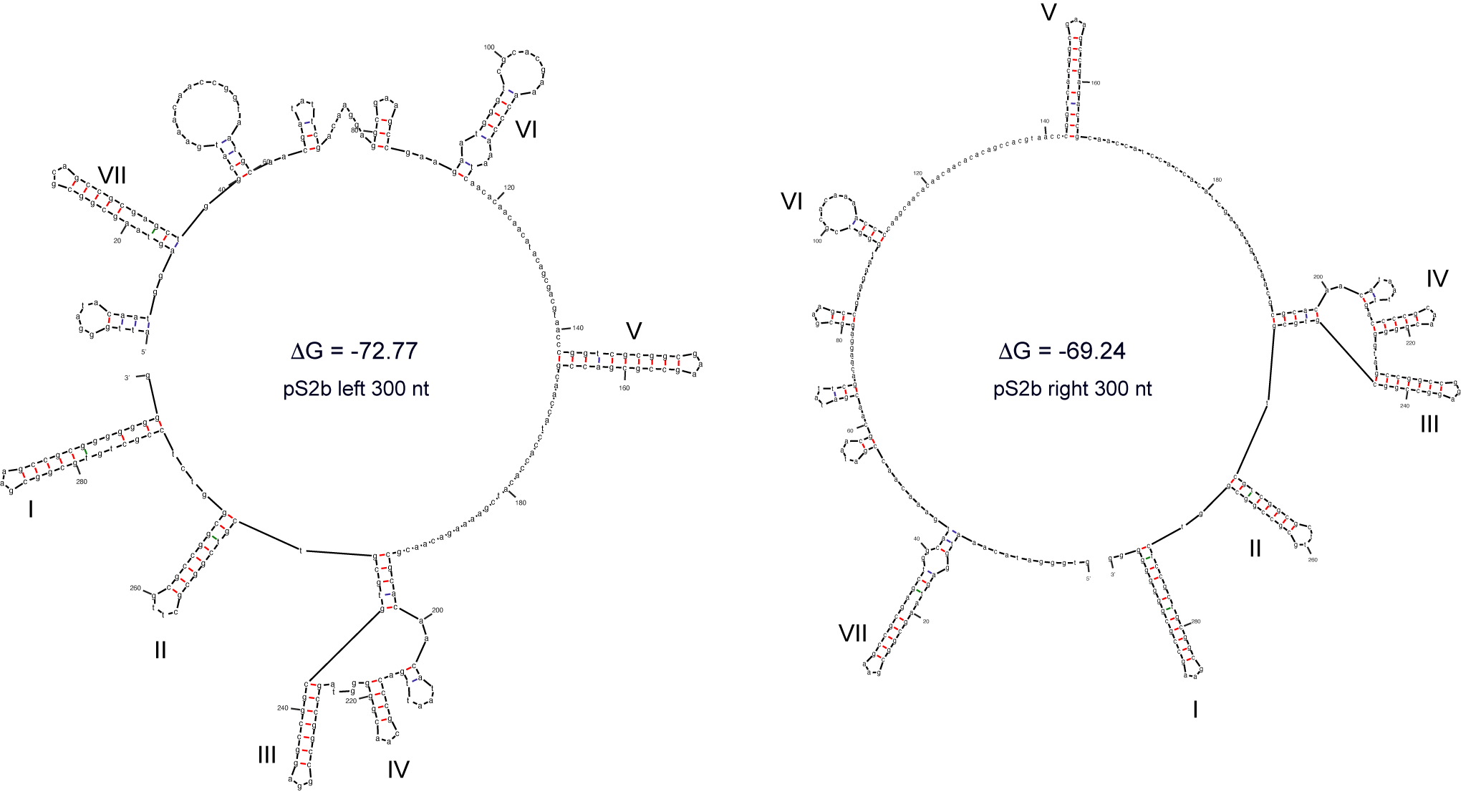


**Fig. S2.** (a) Terminal sequences of pS2b-type linear plasmids. (b) Terminal sequences of pCLP. (c) Folding of complementary strand (lagging-strand template) of pS2b left end 300 nucleotides. (d) Folding of complementary strand of pS2b right end 300 nucleotides. Stem numbers were consistent across all panels. Folding of DNA was predicated under condition of 37 ^o^C 1M Na^+^.
