# Supplementary material for: Giant linear plasmids in Mycobacterium avium harbour a tRNA array unit
Source: DNA Res. 2026 Jan 3;33(1):dsaf039. doi: 10.1093/dnares/dsaf039 (PMC12803027; doi:10.1093/dnares/dsaf039)
Supplement: dsaf039_Supplementary_Data [file dsaf039_supplementary_data.zip › Fig S3.docx]

**
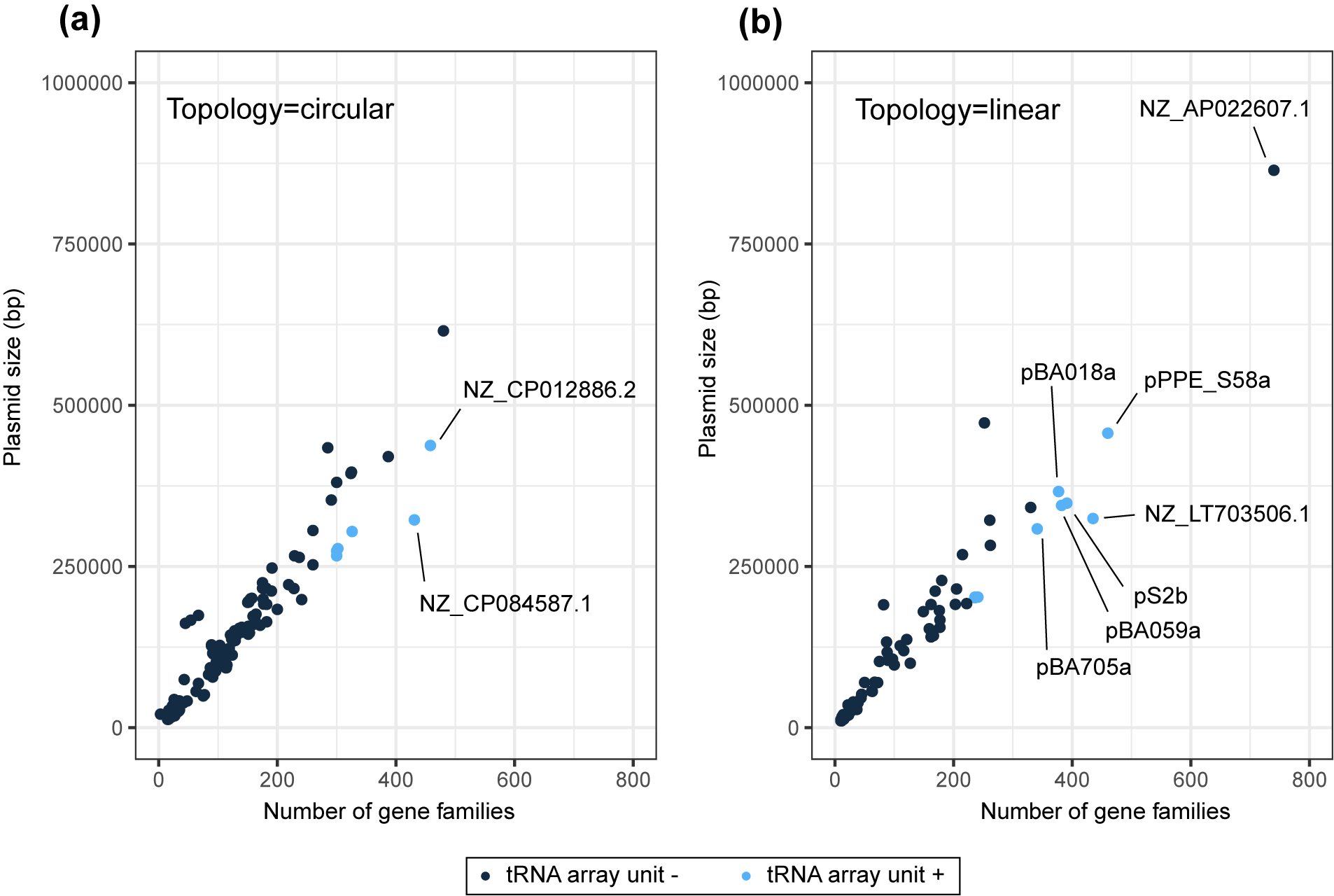
**

**Fig. S3.** Distribution of tRNA array unit-positive plasmids mapped onto the size distribution of NTM plasmids. (a) Circular plasmids (n = 235). (b) Linear plasmids and putatively linear plasmids (n = 79).
