# Supplementary material for: Giant linear plasmids in Mycobacterium avium harbour a tRNA array unit
Source: DNA Res. 2026 Jan 3;33(1):dsaf039. doi: 10.1093/dnares/dsaf039 (PMC12803027; doi:10.1093/dnares/dsaf039)
Supplement: dsaf039_Supplementary_Data [file dsaf039_supplementary_data.zip › Fig S4.docx]

**
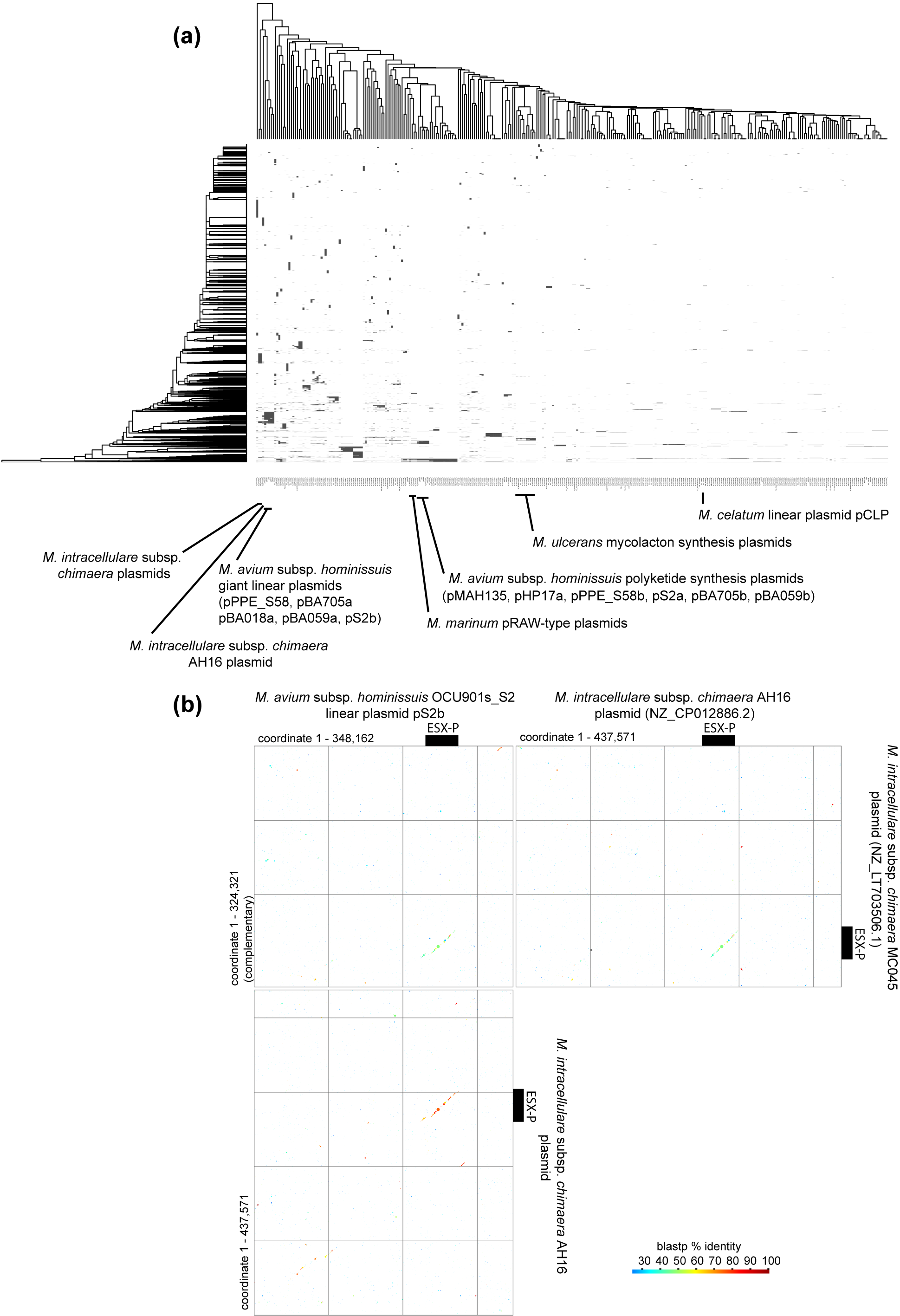
**

**Fig. S4.** (a) Clustering of 314 NTM plasmids based on gene composition. The central subpanel indicates the presence/absence matrix, with plasmids represented on the horizontal axis and 8,336 gene families on the vertical axis. Hierarchical clustering of gene composition dissimilarity was performed using the complete linkage method and Euclidean distance metrics. (b) Comparison of genetic organization between the linear plasmid pS2b and two putative linear plasmids from *M. intracellulare* subsp. *chimera.* Gene synteny similarity is illustrated as two-dimensional plots of the BLASTP results. Sequences were obtained from RefSeq accession numbers NZ_CP012886.2 and NZ_LT703506.1.
