# Supplementary material for: Giant linear plasmids in Mycobacterium avium harbour a tRNA array unit
Source: DNA Res. 2026 Jan 3;33(1):dsaf039. doi: 10.1093/dnares/dsaf039 (PMC12803027; doi:10.1093/dnares/dsaf039)
Supplement: dsaf039_Supplementary_Data [file dsaf039_supplementary_data.zip › Fig S5.docx]

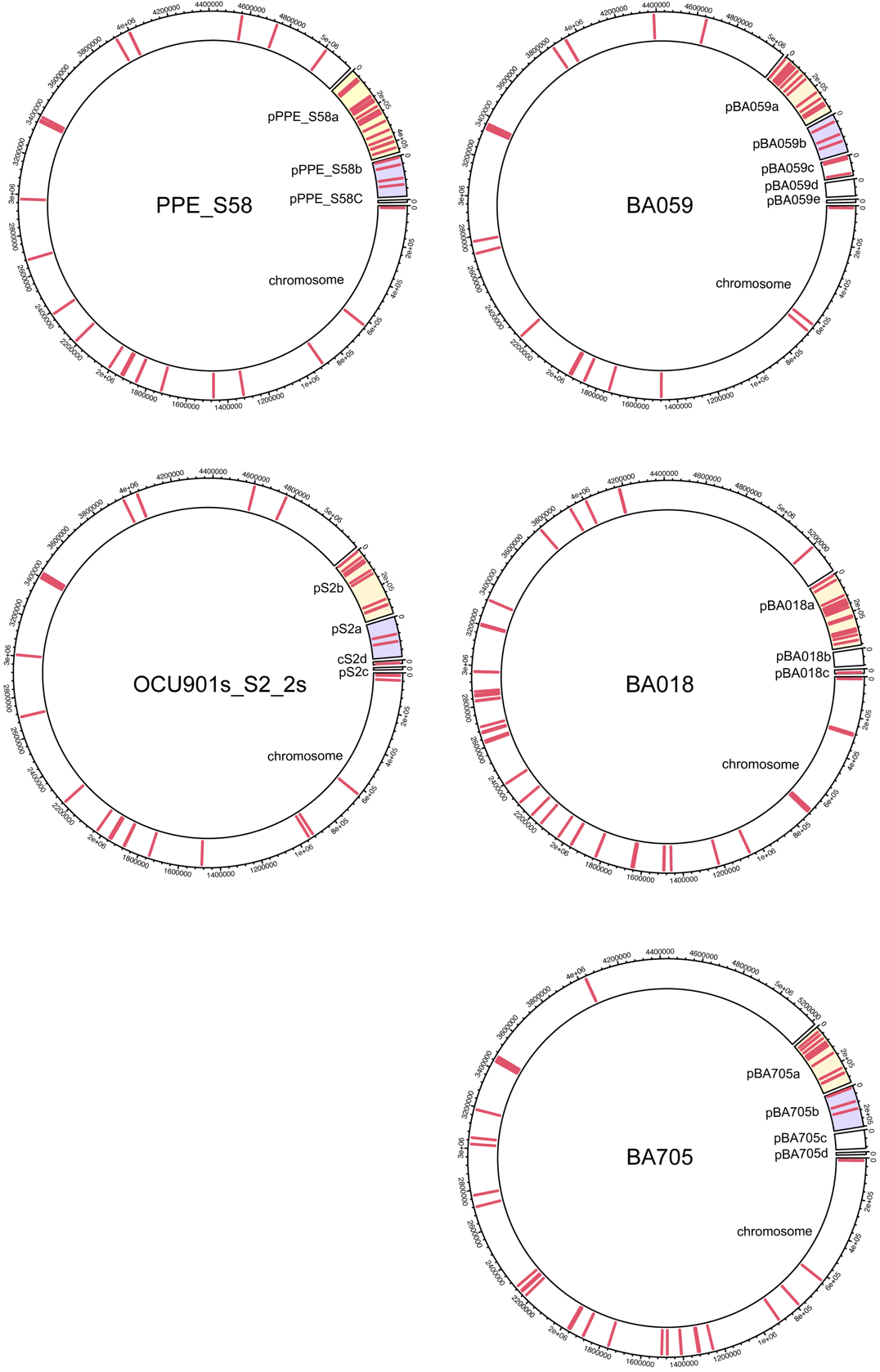


**Fig. S5.** IS locations in the chromosomes and plasmids of five *M. avium* subsp. *hominissuis* strains. All replicons are presented in linear form. IS locations are indicated by red lines. pS2b-type linear plasmids and pMAH135-type putative mycobactin synthesis plasmids are highlighted in yellow and purple, respectively.
